# An Update on New Approaches to Cognitive Assessment in Multiple Sclerosis

**DOI:** 10.3390/neurosci6030087

**Published:** 2025-09-05

**Authors:** Jacob Balconi, Dawn Langdon, Bishal Dhakal, Ralph H. B. Benedict

**Affiliations:** 1 Jacobs Multiple Sclerosis Center, Department of Neurology, Jacobs School of Medicine and Biomedical Sciences, University at Buffalo, The State University of New York, Buffalo, NY 14203, USA; jbalconi@buffalo.edu (J.B.); bishaldh@buffalo.edu (B.D.); 2 Department of Psychology, Royal Holloway University of London, Egham TW20 0EX, UK; d.langdon@rhul.ac.uk

**Keywords:** multiple sclerosis, computerized neuropsychological assessment devices, BICAMS, cognition, neuropsychological tests, psychometrics

## Abstract

Accessible, dependable cognitive assessment is integral to patient care of people with multiple sclerosis (PwMS). Traditional neuropsychological tests are well validated in the multiple sclerosis (MS) population, but not without limitations, such as the time and financial cost associated with traditional in person administration. Recent endeavors have sought to refine assessment, with particular attention to psychometric properties, accessibility, efficiency, and other practical considerations. One approach has been to streamline neuropsychological batteries to brief measures of essential domains, such as the Brief International Cognitive Assessment for MS (BICAMS). Another approach is the use of computerized neuropsychological assessment devices (CNADs). A systematic review of CNADs in PwMS was published in 2019. However, research has continued to expand in the years since. Here, we present an updated review of the BICAMS and further development of CNADs in MS. Tests with strong psychometric foundations are highlighted.

## 1. Introduction

Multiple sclerosis (MS) is an immune-mediated, neurodegenerative disease of the central nervous system (CNS) [1]. Cognitive impairment (CI) is a common symptom, affecting 34–65% of patients [2,3,4]. Slowed information processing speed (IPS) is the most prevalent impairment, although deficits in memory and executive function are common as well [5]. Dependable cognitive assessment is an integral component of comprehensive patient care. CI is linked to occupational outcomes [6] and pre-job-loss factors such as negative work events and the need for workplace accommodations [7]. CI may also impact social cognition and relationships [8], is associated with difficulties in daily functioning [9], reduces quality of life, and impacts social roles [10].

Performance-based assessment of cognitive abilities is necessary due to the multifactorial nature of patient reports in MS [11,12]. Cognitive tests are traditionally administered by a psychologist or psychometrician, and acceptance for clinical use requires strong evidence for reliability and validity. In just the last five years, new tests utilizing modern platforms have emerged that enhance efficiency, accessibility, and other practical considerations. Herein, our goal is to update clinicians and researchers on emerging assessment paradigms. Specifically, we review recent developments in computerized neuropsychological assessment devices (CNADs) and research on the most popular conventional test battery, the Brief International Cognitive Assessment for MS (BICAMS).

## 2. A Traditional Person-Administered Cognitive Assessment—BICAMS

Formative efforts to quantify CI in people with multiple sclerosis (PwMS) were initiated in the 1980s. The first standardized screening tool, or brief battery, was developed in 1990 [13]. However, expert consensus regarding the most valid and cost-effective approach was lacking until the 21st century. In 2002, a panel composed of neurologists and neuropsychologists convened to develop a minimal exam for cognitive monitoring of PwMS. The result, a 90 min battery of seven tests, was the Minimal Assessment of Cognitive Function in MS (MACFIMS) [14]. The MACFIMS was later shown to possess psychometric properties sufficient for broad clinical use [15]. However, its 90 min commitment came to be viewed as burdensome for routine neurological care, leading to calls for a shorter alternative assessment.

In 2012, expert neurologists and neuropsychologists convened to address this issue. The result was the BICAMS, a 15 min cognitive battery [16]. The BICAMS consists of three tests chosen from the MACFIMS: the Symbol Digit Modalities Test (SDMT) [17], and the learning trials from the California Verbal Learning Test—Second Edition (CVLT2) [18] and Brief Visuospatial Memory Test—Revised (BVMTR) [19].

A primary goal of the BICAMS mission was to offer a uniform dataset for multi-country and language research. Toward that end, a validation study protocol [20] was offered soon after the 2012 meeting. Since then, 26 validation studies have been published [21]. As shown in Table 1, the data demonstrate good known-groups validity for all three tests across many cultures. Of the three, the SDMT is the most reliable and sensitive. The BICAMS is consistently associated with employment status [21]. It is noteworthy that these robust psychometric properties allow extensive investigation of MS cognition as applied to, for example, disease phenotypes [22] and brain imaging [23].

Detailed normative values have been published for many countries, beyond the validation studies, including Italy for both adults [50] and adolescents [51], the UAE [52], Brazil [53], Turkey [54] and German-speaking countries [55]. It is essential to take account of national norms [56]. The goal of facilitating international collaboration has been advanced by the BICAMS, with a number of international pharma trials and other research contributions using the BICAMS to collect comparable data from many nations [57].

Many national guidelines recommend the BICAMS for routine clinical assessment, including the American Academy of Neurology Quality Measurement Set for MS [58]. By virtue of the national validations and the convenience of a 15 min battery requiring minimal materials, the BICAMS allows an appropriate and robust cognition assessment to be added to a physical impairment-based clinic assessment. Adding the BICAMS to the Expanded Disability Status Scale (EDSS) increased detection of disease impairment by 10% in a large clinic sample [59].

Because the SDMT is the most-often-used test, and has influenced the growth of CNADs, it deserves its own summary for this paper. The test was first published over 40 years ago [17], but its foundation dates to the early 20th century [60,61]. A version can be found in early test compendiums; it was initially used to screen for learning disability and to measure cognition in children, using a group format administration. The methodology was adopted by the United States Army to screen for cognitive weakness in recruits for World War One. In that version, subjects were instructed to write symbols for numbers, as in the Digit Symbol Coding tests that have served as a processing speed component of the Wechsler intelligence scales [62,63] from the 1930s to the present. In the 2000s, the field began to recognize its utility in MS. It seemed that no matter what was being investigated—sensitivity in discriminating patients from healthy people [15], exploring Magnetic Resonance Imaging (MRI) correlates of cognitive dysfunction [64], or correlation with caregiver reports [11]—it was the most optimal tool available. Soon, it was suggested that it may be a better test than the Paced Auditory Serial Addition Test (PASAT) to screen for cognitive impairment and to serve as the cognitive component of the MS Functional Composite (MSFC) [65,66]. Its popularity has continued to grow to the present. It is used to identify cognitive changes with acute disease activity [67,68] and is commonly included as a secondary outcome in clinical trials [69,70]. It is also a very brief measure, and as such it has been utilized, in one form or another, in many computerized assessments for MS, which are reviewed below.

## 3. CNADs—Update from Wojcik and Colleagues (2019)

CNADs have been studied for decades [71], but their impact has accelerated in recent years. In contrast to traditional person-administered assessments, the computerized platform offers the possibility of self-administration and automated scoring, reducing staff time. A systematic review was published in 2019 by Wojcik et al. [72]. They found 33 individual tests and 11 test batteries that had been applied in PwMS. Strong psychometric properties were found for the following: the Cognitive Drug Research (CDR) battery [73], Cogstate Brief Battery (CBB) [74], NeuroTrax [75], Central Nervous System-Vital Signs (CNSVS) [76], Computerized Symbol Digit Modalities Test (c-SDMT) [77], Processing Speed Test (PST) [78], and Computerized Speed Cognitive Test (CSCT) [79].

To search for CNAD literature published after the Wojcik review, we used the following procedure. First, we conducted individual literature searches on the measures included in the Wojcik review for publications from 2019 or later. We then used the same search strategy cited in the Wojcik paper to find new measures applied in PwMS. Additionally, publications that reviewed [80] or referenced [81] existing tests supplemented our search strategy.

Since publication, the PST, NeuroTrax, and CBB have been utilized in many studies and are reviewed here again. We also cover the Cambridge Neuropsychological Test Automated Battery (CANTAB) [82] and National Institute of Health Toolbox Cognition Battery (NIHTB-CB) [83] due to increased presence in the MS literature. Finally, we review many new tests that have emerged since 2019. The format of administration is summarized in Table 2. As we review the use of CNADs in MS, we should bear in mind that there are many nuances in stimulus presentation, scoring, and normalization/interpretation of raw test scores. There are various degrees of automation and utilization of technicians. The vast majority present visual stimuli and require a motor response, but there are a few exceptions.

### 3.1. NeuroTrax

The NeuroTrax [75] is a technician-assisted computer-based battery of 10 cognitive tests assessing seven cognitive domains. Test instructions are presented on the device screen, although a technician is present to assist as needed. All measures are scored and normed automatically, based on a large normative database (*n* = 1569) [90]. Scores are generated for each domain along with the calculation of a global cognition score (GCS). Different outcomes from the same subtest often contribute to the assessment of multiple domains. For example, on the Stroop task, the word reading trial contributes to the attention score, while the interference component contributes to executive function. Responses are recorded via a mouse. Administration time is approximately 50 min [92]. There were 23 MS-related publications in Wojcik et al. [72], and healthy/MS comparisons of GCS yielded a *d* = 1.1 (subtests ranged from *d* = 0.82 to 1.1). Test–retest reliability coefficients ranged from *r* = 0.40–0.84 in healthy volunteers (HVs), depending on the subtest.

Since 2019, roughly 15 studies have been published. Leach et al. [94] investigated how well SDMT predicts NeuroTrax scores, with modest ability (Receiver Operating Characteristic (ROC) Area Under the Curve (AUC) = 0.623–0.778). Golan et al. [91] assessed correlations between the NeuroTrax and MRI in PwMS. The GCS correlated with structure volume indices of white matter, gray matter, thalamus, lateral ventricles, hippocampus, and lesion volumes. The coefficients ranged from 0.21 for hippocampus volume to 0.46 for whole brain volume. The NeuroTrax has been utilized as an outcome measure in many other recent MS publications [84,89,93,95]. Its psychometric properties are well established; the battery is increasingly used in research studies.

### 3.2. Processing Speed Test (PST)

The PST [78] is an analog of the SDMT included in the Multiple Sclerosis Performance Test (MSPT), a multidomain iPad-based assessment. Alongside the PST are measures of walking speed, balance, upper extremity mobility, and visual acuity [109]. The PST presents one row of number–symbol pairings at the top of the screen. Participants respond by pressing one of nine number keys at the bottom. The test may be administered with or without technician assistance. Performance in both conditions has shown high agreement (i.e., similar means) in PwMS (*p* = 0.193) and HVs (*p* = 0.691) [78]. Subsequent investigations have supported the validity of self-administration [106,108]. The PST is also scored and normed automatically. Furthermore, the number–symbol pairings are randomly generated from a pool of 30 symbols with each administration, reducing learning effects in repeated administrations [78].

Three MS-related publications on the PST were reviewed in Wojcik et al. [72], yielding insight into test–retest reliability (Concordance Correlation Coefficient (CCC) = 0.88), known-groups validity (MS vs. HV) (*d* = 0.75), and concurrent validity with the SDMT (*r* = 0.75–0.80). Recommendations for cognitive screening listed the PST as an IPS measure sufficiently validated to use as an alternative to the SDMT in routine clinical care [168].

Publications on the PST were limited in 2019, while applications in PwMS have surged in recent years. Hechenberger et al. [101] compared SDMT and PST correlations with MRI, the BICAMS, and psychological variables, finding each measure’s correlations were largely comparable. The authors also demonstrated the feasibility of self-administration in waiting rooms, with performance in waiting rooms and quiet testing rooms displaying no difference (*p* = 0.970). Although multiple recent studies have found significant discrimination between PwMS and HVs, substantially fewer PwMS were classified as CI by the PST (5.2%) than the SDMT (27.3%), leading authors to conclude the typical cut-off score of the current PST (−1.5 SD) may not be appropriate in Europe [101]. In contrast, Galioto et al. [100] administered a battery of eight traditional neuropsychological tests, detecting impairment in 42.9% of PwMS. As might be expected, the impairment frequency for PST was 23.1%, compared to 20.9% for the processing speed component of the traditional battery.

Jaworksi III et al. [102] compared the ability of the PST and SDMT to predict employment deterioration. Analysis of covariance (ANCOVA) models first compared HVs, employed PwMS, and disabled PwMS on the PST (Eta squared = 0.113, *p* < 0.001) and the BICAMS (Eta squared = 0.034–0.105, all *p* < 0.001). Post hoc analyses revealed all measures distinguished disabled PwMS from employed PwMS and HVs. However, the PST was the only measure that discriminated employed PwMS from HVs. Two years later, two groups were compared: work-stable; work-deterioration. Using baseline measures, these groups were significantly different on both the SDMT (*d* = 0.603, *p* = 0.005) and the PST (*d* = 0.610, *p* = 0.003). A logistic regression model favored the PST over the SDMT, but the effects were similar for each test. Similar employment findings were observed by Macaron et al. [105], who also found significant PST/MRI correlation (whole brain *ρ* = 0.50). The PST also correlated with patient-reported outcomes (PROs) measuring quality of life (QoL).

As of 2019, one weakness of the PST was the lack of a large normative database. Such is no longer the case. Regression-based norms (*n* = 428) are available and are in the device normalization [107]. Furthermore, the PST is included in the Multiple Sclerosis Partners Advancing Technology and Health Solutions (MS PATHS) project and had been administered to 18,001 PwMS as of 2021 [107]. PST data collected as part of the MS PATHS initiative have led to numerous publications utilizing the test as the primary cognitive outcome in many studies [96,99,103,104].

### 3.3. Cogstate Brief Battery (CBB)

The CBB [74] is a brief iPad or computer-based battery of four tests measuring visual learning, working memory, attention, psychomotor function, and IPS. Responses are recorded via an on-screen interface presenting “yes” and “no” buttons. Each test utilizes playing cards, asking the examinee a different question for each subtest (e.g., “Have you seen this card before?”). In two subtests the primary outcome is reaction time (Detection Test (DT) and Identification Test (IT)), while accuracy is primary in the remaining two (One Card Learning Test (OCLT) and One Back Test (OBT)). Subtests are automatically scored and normed with each administration. Test administration may be unsupervised, with unsupervised administration recently validated in a large sample (*n* = 19,476) [110]. Recent comparisons of at-home and in-clinic administrations in a large sample of healthy older adults revealed mostly similar, albeit sometimes different, performances; participants were less accurate on OCLT and OBT and marginally faster on DT when performing at home [117]. However, an earlier study of PwMS (*n* = 80) and HVs (*n* = 28) found no differences between technician-present and technician-absent conditions [118].

Five MS-related publications using the CBB were reviewed in 2019. In HVs, reliability coefficients ranged from 0.31 to 0.94, depending on the subtest and intertest interval. CBB variables discriminated PwMS from HVs, with effect sizes ranging from *d* = 0.41 to 0.79. Since then, the range of measures has expanded and the intraindividual variability (IIV) of reaction time (RT) has proven to be a sensitive measure of CI [116].

Eilam-Stock et al. [112] compared the CBB, the BICAMS, and two other measures (Attention Network Test—Interaction (ANT-I) and Test of Everyday Cognitive Ability (TECA)) in their ability to detect early cognitive involvement in PwMS. Participants included early-stage PwMS along with demographically matched HVs. ROC analyses predicting diagnosis (MS vs. HV) revealed CBB IT (AUC = 0.73, *p* = 0.004), ANT-I IIV (AUC = 0.79, *p* = 0.001), and TECA (AUC = 0.78, *p* = 0.001) to be the most sensitive and specific measures. However, none of the BICAMS measures met the *p* < 0.01 significance threshold (*p* = 0.03–0.28). CBB RT IIV data were collected; however, results were nonsignificant (*p* = 0.10). With a large normative sample, suitability for self-administration, comprehensive study in other populations, and substantial study in PwMS [111,113,114,115], the CBB shows growing promise as a cognitive measure for PwMS.

### 3.4. Cambridge Neuropsychological Test Automated Battery (CANTAB)

The CANTAB [82] is a technician-assisted battery with many subtests that may be administered on a computer or an iPad [121,122]. The battery measures multiple domains. Those commonly assessed in PwMS are episodic memory, visuospatial retention/manipulation, IPS, and sustained attention. Wojcik et al. [72] reported that test–retest reliability coefficients in older adult HVs ranged from *r* = 0.56 to 0.86, although these data were collected for only five tests. Effect sizes discriminating PwMS from HVs was reported for 10 subtests, ranging from *d* = 0.22 to 1.38.

A recent publication by Karlsen et al. [120] set out to update psychometric research on nine of the most used tests, not specific to MS research. Among 75 HVs, retest reliability coefficients ranged from *r* = 0.39 to 0.79, in line with previous research. Reliable change indices required large score changes, ranging from one to nearly two standard deviations, depending on the measure. A paper by Talebi et al. [122] utilized an atypical cross-sectional study that included only PwMS. Curiously, they concluded that both MACFIMS and a four-subtest version of the CANTAB had equal validity, despite no direct comparisons in discriminating patients and HVs. Their data do show a fair to good correlation between the batteries and large effect sizes when comparing CI PwMS and non-CI PwMS (*d* = 1.1–1.6). Furthermore, a similar four-test version of the CANTAB, emphasizing visual memory, discriminated between relapsing and stable patients and showed recovery of function at 1- and 3-month intervals [119].

### 3.5. National Institute of Health Toolbox Cognition Battery (NIHTB-CB)

The NIHTB-CB [83] is a tablet-based cognitive battery. Fluid cognition measures (approximately 30 min administration) [124] include five tests, emphasizing IPS, working memory, episodic memory, and executive functioning, reduced to a fluid cognition composite score. The battery’s interface presents instructions to the examinee, who records responses via touchscreen or an oral response recorded by the examiner. Measures are scored and normed automatically. A new version (V3) was released in 2024, including updated psychometric properties and normative data [123]. Test–retest reliability of the updated NIHTB-CB was assessed in 190 HVs. Fluid cognition subtest reliability coefficients in HVs ranged from *r* = 0.71 to 0.81. In the Wojcik et al. [72] review, there was just one MS-related publication utilizing only one subtest, the Modified Flanker Task, a measure of cognitive speed and inhibitory control. Data were presented on test–retest reliability in HVs (intraclass correlation coefficient (ICC) = 0.83), known-groups validity (*d* = 0.60–0.84), and concurrent validity with the Delis–Kaplan Executive Function System (D-KEFS) Color–Word Interference Test (*r* = 0.48).

Since then, in a study from the Prakash group [124], 87 PwMS were evaluated with the MACFIMS, selected tests from the Wechsler Adult Intelligence Scale (WAIS) and the fluid cognition NIHTB-CB subtests: Pattern Comparison, List Sorting, Picture Sequence Memory, Dimensional Change Card Sort, and Flanker Test. Convergent validity coefficients ranged from *r* = 0.23 to 0.53. Classifications of abnormality were also similar for the NIHTB-CB and the MACFIMS. The authors recommended that the NIHTB-CB shows promise as a cognitive screener in MS. The NIHTB-CB has a robust normative database, strong evidence of test–retest reliability in HVs and promising preliminary known-groups validity results. However, future research in PwMS, particularly regarding detection of CI, is necessary.

## 4. CNADs—New Tests Applied in MS After 2019

Many new measures have been used in PwMS since the 2019 Wojcik et al. [72] publication. Given their recent emergence, most measures have seen limited study. Here, we briefly review these measures.

### 4.1. Auditory Test of Processing Speed (ATOPS)

The ATOPS [126] is a technician-administered smartphone-based measure of IPS. The test was developed to avoid the visual confounds of the SDMT, the stress of the PASAT, and the involvement of visual processing, motor response, and, to a large extent, working memory. There is no episodic memory required, and the working memory demands are minimal. Patients respond with only a yes or no response, minimizing complex oral speech and language function. The yes/no response follows presentations of single- or double-digit numbers, presented aurally, in response to rules of increasing complexity: [a] “Was the number above/below 50?” and [b] “Is the number above/below 50 or an even/odd number”? The test was designed as a computerized assessment, taking advantage of the smartphone medium; stimuli are presented by a standardized artificial voice, and response times are calculated by the app.

The ATOPS is still in the developmental stages. Designed for use in severely disabled MS patients, including those with EDSS > 7 and even those who are “locked-in” in some cases, the completion rate of the ATOPS far exceeded that of the SDMT [126]. There was no correlation with maximum repetition rate (MRR) [125], which was modestly correlated with the PASAT (*r* = 0.31), the SDMT (*r* = 0.29), and verbal fluency (*r* = 0.31) in the same sample. The ATOPS was predicted by thalamic volume, effectively discriminated nursing facility PwMS from community-dwelling PwMS (*d* = 1.07), and showed a higher completion rate (93.2%) than the SDMT (65.9%) in severely disabled patients [125]. Weinstock et al. [126] reported large effect sizes (*d* = 0.74–0.86) in discriminating between PwMS and HVs, correlations with the SDMT (*r* = −0.53 to −0.59), discrimination between CI PwMS and non-CI PwMS (*d* = 0.62–0.78), correlation with patient lesion burden (*r* = 0.33–0.44), and a higher completion rate in severely affected people with MS (SAPwMS) than the SDMT (72% vs. 100%).

After observing the test’s preliminary promise, a new version of the ATOPS was developed. Improvements included repeatable practice rounds (to facilitate task comprehension), redesignation as an iPhone operating system (iOS) application (enhancing measurement by removing internet reliance), and the creation of an alternate form (allowing for assessment of test–retest reliability). Examinees’ oral responses are recorded by administrators, whose reaction times are calibrated and subtracted from the total time. However, an additional upgrade to the ATOPS, adding automated speech recognition to enhance measurement, is currently being investigated.

### 4.2. Adaptive Cognitive Evaluation (ACE)

ACE is a technician-assisted tablet-based battery, measuring attention, IPS, memory, and executive function across four subtests [127]. Responses are recorded via a touchscreen interface. Administration is generally self-guided, although a technician is present to answer any questions. Each subtest utilizes a similar procedure: A target is presented until a response is received or the reaction time limit is reached. Hsu et al. [127] published an initial application in PwMS. Analysis of variance (ANOVA) models found some ACE measures (i.e., reaction time and attention) distinguished HVs and non-CI PwMS from CI PwMS (*p* < 0.05) while correlating with the SDMT (*r* = −0.57) and the PASAT (*r* = −0.39) [127].

### 4.3. EVO Monitor

The EVO Monitor is an iPad-based measure of attentional control and other related aspects of cognition [128]. The measure is designed as an immersive action video game with dynamic difficulty based on performance. Participants use the iPad to navigate and defend a character through a series of obstacles, taking about 7 min to complete. The measure consists of three tasks: perceptual discrimination, visuomotor tracking, and multitasking, each scored automatically. Hsu et al. [128] published an initial application in MS, showing the EVO Monitor correlated with the SDMT (*r* = 0.52), walking speed (*r* = −0.45), and brain volumetric data (*r* = 0.27–0.47). ANCOVA models revealed worse performance in CI PwMS compared to non-CI PwMS and HVs on all three outcomes (*p* ≤ 0.01). A clinical trial administering the EVO Monitor once every two weeks is now underway [129].

### 4.4. iCognition

iCognition [81] is a smartphone-based, examiner-supervised cognitive screening battery. It consists of three subtests assessing IPS (Symbol Test), working memory (Visual Backward Digit Span (vBDS)), and short-term visuospatial learning and memory (Dot Test (similar to the Spatial Recall Test (SPART), also known as the 10–36 test)) [13]. Responses are recorded via the touchscreen interface. The Symbol Test is similar to the SDMT, albeit somewhat different; participants view a key of nine symbol–symbol pairs at the top of the screen. When another pairing appears below, the examinee presses yes or no to specify whether the presented pair matches any in the key. Subtests correlated well with pencil–paper equivalents for the vBDS and Symbol Test (*r* = 0.69 and 0.67) [81]. Test–retest reliability coefficients ranged from ICC = 0.71 to 0.74 (*r* = 0.74–0.85). However, the MS sample showed no signs of impairment, as no differences were found between PwMS (*n* = 101) and HVs (*n* = 82) on pencil–paper (*p* = 0.37–0.82) or iCognition (*p* = 0.27–0.42) measures.

### 4.5. Symbol Search and Dot Memory Ambulatory Cognitive Tests

Ambulatory cognitive tests are smartphone-/iPad-based digital cognitive assessments, where patients are tested multiple times per day outside the clinic. Two such measures were utilized in a 2025 study [130], which have been validated in non-MS populations [132,133]: Symbol Search (IPS measure) and Dot Memory (working memory). The Dot Memory test is similar to the SPART, and in fact inspired the Dot Test of iCognition. Symbol Search is administered by displaying four symbol–symbol pairs at the top of the screen and two symbol–symbol pairs at the bottom; examinees select which of the bottom pairs is present in the top array. Goga et al. [130] published a feasibility study administering the tests in PwMS 4×/day. The results demonstrated good adherence (80.1% completion of testing sessions), and a 14-day reliability estimate model showed good results. A large study administering the Symbol Search and Dot Memory tests 4×/day alongside the NIHTB-CB and traditional measures is currently underway (CogDetect-MS Study) [131].

### 4.6. Virtual Reality Attention Tracker (VRAT)

The VRAT [135] is a virtual reality program used to assess attention by embedding a continuous performance task (CPT) in a virtual classroom. A controller is used for participants to record their responses. Hsu et al. [134] applied the measure in PwMS, showing the SDMT correlated with VRAT reaction time variability, both with (*r* = −0.67) and without (*r* = −0.57) distractors. Additionally, reaction time variability was significantly different between PwMS and HVs (*η* ^2^ = 0.23–0.29, *p* = 0.01–0.03).

### 4.7. Brief Assessment of Cognitive Health (BACH)

The BACH is a self-administered iPad-based assessment that presents auditory test instructions through headphones [137]. This 20 min battery has two cognitive subtests, both assessing memory with immediate and delayed recall components: the verbal memory subtest (words) and the nonverbal memory subtest (faces). The BACH compared favorably with the SDMT when discriminating between CI (as defined by performance < 5th percentile on any traditional measure) and non-CI PwMS (BACH AUC = 0.78; SDMT AUC = 0.73) and identifying non-IPS impairment (BACH AUC = 0.71; SDMT AUC = 0.56) [137]. Furthermore, Floden et al. [136] conducted a large validation study in HVs (*n* = 250) and a general clinical sample of patients referred for neuropsychological testing (*n* = 440). Test–retest reliability in HVs was assessed by the total proportion of correct responses across both subtests (*r* = 0.70). BACH measures largely correlated with various traditional neuropsychological measures in HVs.

### 4.8. MSReactor

MSReactor is a self-administered web-based cognitive assessment battery that may be administered at home or in clinic [138]. Three subtests measure psychomotor processing speed, visual attention, and working memory. Psychomotor processing speed is assessed by a simple reaction time task, visual attention is indexed by a choice reaction time task, and working memory is examined by a one-back task. Test–retest reliability was assessed over nine administrations [138]. Reliability coefficients for the first two administrations ranged from CCC = 0.49 to 0.68 but increased when comparing administrations 8 and 9 (CCC = 0.81–0.86). Each subtest correlated with the SDMT (*r* = −0.43 to −0.59) and discriminated PwMS from HVs after controlling for education (*p* ≤ 0.02). Additional studies found MSReactor measures to be correlated weakly with subjective cognitive complaints (|Tau| = 0.10–0.17) [139] and correlated moderately with the PST (*r* = −0.40 to −0.47) [140].

### 4.9. Integrated Cognitive Assessment (ICA)

The ICA is a self-administered 5 min IPS test that presents photos of animals and non-animals in rapid succession on an iPad [141]. Examinees use the touchscreen to record whether each stimulus is an animal or a non-animal as quickly as they can. Response accuracy and speed are collected and used to calculate a summary score (ICA score). The ICA then uses an artificial intelligence system to generate a predicted cognitive status based on demographic data, accuracy, speed, ICA score, and other testing characteristics; this report is automatically generated and can be delivered to an electronic health record. Khaligh-Razavi et al. [141] published an initial validation study in PwMS and HVs. The ICA score distinguished HVs from PwMS (*d* = 1.26, *p* < 0.001) and demonstrated correlations with the SDMT (*r* = 0.71–0.81), the BVMTR (r = 0.54–0.80), and the CVLT2 (r = 0.58–0.76) in PwMS and HVs. Furthermore, ROC analysis revealed that the ICA score effectively detected CI defined by the BICAMS (AUC = 0.951). Test–retest reliability coefficients were excellent in PwMS (*r* = 0.94) and HVs (*r* = 0.91). However, the known-groups validity did not replicate in a follow-up study of cognition and MRI metrics in relapsing–remitting MS (RRMS) patients [142]. Despite decreased thalamic volume in RRMS patients (relative to HVs), there was no significant difference between PwMS and HVs on reaction time.

### 4.10. Brain on Track (BoT)

BoT is a self-administered computer-based battery of 11 tests assessing domains such as attention, IPS, memory, executive function, language comprehension, and visuospatial ability along with a total score (BoT score) [143]. The battery is typically completed in 24 min. Ruano et al. [143] published an initial validation study in PwMS after the battery was developed in other populations [144,145]. The BoT score (*d* = 0.87, *p* < 0.01) and 9 of 11 subtests (*d* = 0.52–0.99) distinguished HVs from PwMS. PwMS were classified as CI or non-CI via a standard neuropsychological evaluation including the BICAMS, some WAIS tests, Stroop tasks, and others. The BoT score distinguished the groups (*d* = 2.0) and demonstrated a significant AUC (0.91). All 10 tests from the standard neuropsychological battery correlated with the BoT score (*r* = 0.43–0.71). Test–retest reliability coefficients included all four administrations (1 supervised, 3 at home), with 9 of 11 subtests demonstrating coefficients > 0.80 (ICC = 0.60–0.90).

## 5. New Computerized Variants of the BICAMS

Due to the popularity of the BICAMS and the benefits of CNADs, multiple computerized variants of the BICAMS have recently been developed.

### 5.1. Multiple Screener

The Amsterdam Vrije Universiteit group developed a brief iPad battery dubbed the Multiple Screener [146,147] intended to be analogous to the BICAMS. The test is self-administered. It includes a Dutch translation of the CVLT2, the SDMT, and the SPART. The SPART was chosen over the BVMTR due to its suitability for automated scoring. For the CVLT2, participants type each word. For the SDMT, participants type the correct digit in the space below each symbol using the number pad. The SPART displays a 36-square grid with 10 black checkers, followed by an empty grid onto which participants swipe checkers into the correct locations. Van Dongen et al. [146] provided regression-based norms (*n* = 225–235) and demonstrated strong agreement between computerized and traditional versions of the SDMT (ICC = 0.79), the CVLT2 (ICC = 0.77), and the SPART (ICC = 0.61). Furthermore, Waskowiak et al. [147] recently published the study protocol for a large validation study (*n* = 750 PwMS), which is currently underway.

### 5.2. iCAMS

Beier et al. [148] developed a technician-administered tablet-based method for the BICAMS, called the iCAMS. The Rey Auditory Verbal Learning Test (RAVLT) was used instead of the CVLT2. Responses were recorded on an iPad device using a platform developed by the authors. For the SDMT, the standard paper stimulus was presented, and responses were recorded using the iPad. The RAVLT word list was read aloud by the administrator who recorded responses via touchscreen. The BVMTR stimuli were presented on the iPad, and the 10 s presentation was automatically turned off, followed by a blank screen. Participants rendered the figures from memory using their finger or a stylus. The platform eased scoring, as the RAVLT and SDMT scores were calculated automatically, and the tablet displayed the BVMTR responses next to the scoring criteria. These administration methods resulted in scores correlating with standard methods (ICC > 0.93). Furthermore, full administration and scoring of the iCAMS was 10 min shorter than the standard BICAMS [148].

### 5.3. Brief Computerized Cognitive Assessment for MS (BCCAMS)

Maubeuge et al. [149] developed a technician-supervised computer-based battery called the Brief Computerized Cognitive Assessment for MS (BCCAMS). The first subtest is the CSCT, a computerized analog of the SDMT reviewed in Wojcik et al. 2019 [72]. The CSCT is included as a validated SDMT alternative for routine neurological screening in the 2018 guidelines endorsed by the Consortium of Multiple Sclerosis Centers (CMSC) and the International Multiple Sclerosis Cognition Society (IMSCOGS) [168]. Alongside this measure is the Computerized Episodic Visual Memory Test (CEVMT), which presents a 4 × 4 matrix for 25 s, forming a complex figure of contiguous squares and triangles. Like the BVMTR, three trials are administered whereby the examinee attempts to reassemble the figure from memory. The third measure is the French Learning Test (FLT), the verbal episodic memory measure of the French BICAMS. However, the FLT was administered in traditional paper-based format. Regression-based norms were developed (*n* = 276), which were used to establish a definition of CI (z-score < −1.5). The BCCAMS detected CI in 59% of PwMS, which was higher than the BICAMS (50%) and the MACFIMS (38%). All subtests distinguished PwMS and HVs (*d* = 0.47–0.82). Test–retest reliability of the CEVMT was assessed in PwMS (*r* = 0.70) and HVs (*r* = 0.60); the CEVMT also correlated with the BVMTR (*r* = 0.51–0.52) [149]. However, the authors hope to improve the test–retest reliability with future development of alternative forms and are currently validating a computerized version of the FLT.

### 5.4. iBICAMS

Costabile et al. [150] developed a technician-administered iPad version of the BICAMS, named the iBICAMS. The BVMTR is administered in a similar fashion to the iCAMS. The stimuli are presented for 10 s followed by a blank screen used by examinees to draw their responses (via stylus). The CVLT2 plays a recording of the word list and presents a dropdown menu for the examiner to highlight patient responses. The battery uses a paper stimulus for the SDMT, while the administrator records responses on the iPad. The BVMTR is scored by the administrator, while the CVLT2 and the SDMT are scored and normed automatically. All iBICAMS measures showed high agreement with paper–pencil equivalents (ICC = 0.82–0.92), although paired-samples t-tests found raw scores differed between the BICAMS and the iBICAMS (*p* ≤ 0.015).

### 5.5. Digital Assessment of Cognitive Impairment in Multiple Sclerosis (DIGICOG-MS)

The DIGICOG-MS [151] is a smartphone or tablet-administered assessment with four subtests (Remember and Place (visuospatial memory), Listen and Repeat (verbal memory), Generate Words (semantic fluency), and Associate Numbers (IPS)). Subtests are based on traditional measures such as the SPART, the RAVLT, the SDMT, and Word List Generation (WLG). However, two subtests require a technician to record and/or score responses, as there is no voice recognition by the computer or device. All four measures demonstrated good concurrent validity with corresponding traditional measures (*r* = 0.58–0.78). DIGICOG-MS subtests demonstrated good test–retest reliability, with coefficients ranging from ICC = 0.83 to 0.96, depending on the subtest.

## 6. New Computerized Variants of the SDMT

Current recommendations for cognitive screening of PwMS suggest, at minimum, annual screening using the SDMT or a similarly validated measure [168]. In these recommendations, the PST and the CSCT are explicitly mentioned as validated SDMT alternatives. Although not mentioned, the c-SDMT has also previously demonstrated promising results [72]. Next, we look at other examples of emerging measures we consider to be analogous to the SDMT—that is, SDMT variants.

### 6.1. Konectom Cognitive Processing Speed (CPS) Test

The Konectom CPS test is a smartphone-based, self-administered test designed to assess cognitive processing speed in clinical and daily living environments [152]. Test administration may be supervised (i.e., in clinic) or unsupervised (i.e., at home). The measure includes two tasks: symbol-to-digit (S2D) and digit-to-digit (D2D). The S2D is similar to the SDMT (examinees select numbers for corresponding symbols for 90 s). Measures were collected across seven administrations [152]. S2D test–retest reliability coefficients ranged from ICC = 0.80 to 0.87. Each S2D outcome also correlated with the SDMT, whether administered in clinic (|*ρ*| = 0.72–0.77) or at home (|*ρ*| = 0.79–0.80). Known-groups validity was strong, with S2D effect sizes ranging from *d* = 1.29 to 1.47 in distinguishing CI from non-CI PwMS (as defined by z-score < –1.5 on any single BICAMS test).

### 6.2. elevateMS

elevateMS [153] is a smartphone-based app designed to remotely capture real-world MS-related health data. The app includes many measures, including demographic data, upper-/lower-body mobility, cognitive function, local weather data, and PROs (e.g., physical ability, MS symptoms/triggers, mobility, pain, and the short-form Quality of Life in Neurological Disorders (Neuro-QoL)). The sole cognitive measure is the voice-based Digit Symbol Substitution Test (DSST). Participants use the microphone to announce their response to each item. The group has reported correlations between the test and Patient-Determined Disease Steps (PDDSs) and QoL PROs, but also reports that the HV data were discarded due to low compliance [153].

### 6.3. Smartphone-Adapted SDMT (sSDMT)

The sSDMT is a smartphone variant of the conventional SDMT embedded in the MS Sherpa smartphone application that may be self-administered [155]. One symbol is presented at a time, with responses recorded via a touchscreen interface. Van Oirschot et al. [155] conducted an initial validation study where participants completed the sSDMT at home once every three days for four weeks. The first administration of the sSDMT demonstrated a difference between PwMS and HVs closely matched on age, gender, and education (*d* = 0.73, *p* = 0.02) [155]. Lam et al. [154] conducted a similar validation study, finding test–retest coefficients to be ICC = 0.88. Furthermore, sSDMT administrations correlated with the SDMT (*r* = 0.62–0.69), the CVLT2 (*r* = 0.45–0.49), the BVMTR (*r* = 0.53–0.56), and the EDSS (*ρ* = −0.48 to −0.42). The sSDMT also distinguished CI (as defined by SDMT) from non-CI PwMS (AUC = 0.922, *p* < 0.001), non-CI PwMS from HVs (AUC = 0.639, *p* = 0.044), and all PwMS from HVs (AUC = 0.713, *p* = 0.001) [154].

### 6.4. Floodlight Open

Floodlight Open is a smartphone app-based remote assessment of PwMS [157]. The app collects many measures (e.g., motor function, gait, and PROs), although two tests represent the sole cognitive outcomes: an electronic implementation of the SDMT (information processing speed (IPS) test, sometimes referred to as e-SDMT or electronic implementation of the SDMT) and a digit-to-digit matching task (IPS Digit-Digit (IPS DD)) [157,158,159,160]. Both measures are administered via the smartphone’s touchscreen interface, where examinees select their responses. The IPS test is the primary cognitive measure, while the IPS DD is sometimes applied for additional insight into the influence of motor/visual functioning on IPS scores. Measures collected via Floodlight Open are completed frequently, with cognitive measures typically completed weekly. Studies have shown satisfying engagement with the Floodlight Open measures [157], which was further improved with additional user interface features [156]. A 24-week validation study provided test–retest reliability estimates (HV ICC = 0.55; PwMS ICC = 0.85) and correlation with the SDMT (*ρ* = 0.82), the EDSS (*ρ* = −0.43), the 29-item Multiple Sclerosis Impact Scale (MSIS-29) (*ρ* = −0.52), T2-weighted Fluid-Attenuated Inversion Recovery (T2-FLAIR) lesion volume (*ρ* = −0.42), and normalized brain volume (*ρ* = 0.54) [158]. Furthermore, a 2024 study found that the IPS test discriminates PwMS from HVs (controlling for age and sex), albeit with very small effects (*d* = 0.054–0.083) [159].

### 6.5. Electronic SDMT (eSDMT)

The eSDMT was developed in 2025 by Dini et al. [161]. The test is administered on a computer via an internet browser and presents symbols one at a time with responses recorded via a keyboard. Examinees may complete the test at home. Alongside the eSDMT is the Numbers task, where examinees are presented with a single-digit number and must select the same number via keyboard. Unlike the SDMT, both measures utilize reaction time as the primary outcome, with a fixed number of stimuli presented per administration (eSDMT: 54 stimuli; Numbers: 27 stimuli). eSDMT RTs correlated strongly with oral SDMT administration (*r* = 0.87) and demonstrated strong test–retest reliability (ICC = 0.88). Known-groups validity was assessed by breaking the MS sample into CI and non-CI groups (via BICAMS definitions) followed by ROC analyses predicting cognitive status (AUC = 0.846, *p* < 0.0001).

### 6.6. Cognition Reaction (CoRe)

CoRe is a tablet-based assessment developed by Middleton et al. [162]. The measure presents symbols with their corresponding numbers (1–9) at the top of the screen, with an additional row of numbers presented at the bottom for participants to record responses. Examinees are presented with two items at a time (i.e., the current item and the next item). The primary outcomes are the number of correct responses and RTs for individual items. The number of correct responses distinguished PwMS from HVs (*d* = 1.21, *p* < 0.001), correlated with the written SDMT (*r* = 0.80), and demonstrated excellent test–retest reliability (ICC = 0.94).

### 6.7. Mobile Cognition Test (MCT) (MSCopilot)

The MCT [163] is the cognitive measure within MSCopilot, a smartphone application measuring walking, dexterity, cognition, and low-contrast vision. Items are presented one at a time, with responses recorded via a touchscreen interface. The MCT demonstrated a test–retest reliability coefficient of ICC = 0.74 [163] and distinguished PwMS with EDSS > 3.5 from those with 3.5 or lower (*p* = 0.009) [164]. However, most analyses combine MSCopilot measures into a composite score, comparing results with the MSFC.

### 6.8. Modified SDMT (MD-SDMT)

Seo et al. [165] developed the MD-SDMT, a tablet-based SDMT. A symbol–digit key is presented at the top of the screen. Participants use the touchscreen interface to select the number for each symbol, like many computerized SDMTs. The MD-SDMT has two forms: one-minute administration and two-minute administration. The authors recruited a sample of PwMS and neuromyelitis optica spectrum disorder (NMOSD) patients. MD-SDMT performance was compared to written SDMT administration (*r* = 0.85–0.88), with patients generally preferring the MD-SMT over the traditional paper-based administration.

### 6.9. Smartphone-Based SDMT (NeuFun)

Pham et al. [166] developed a smartphone-based SDMT to be included in their Neurological Functional Test Suite (NeuFun), a patient-autonomous smartphone app designed to capture essential domains of neurological examination. This SDMT adaptation presents only one symbol at a time. A touchscreen number keypad is presented at the bottom of the screen for participants to record their responses. The authors also developed an oral response version of their measure utilizing vocal recognition technology to avoid motor confounds. However, the patients were allowed to choose between touchscreen and vocal administrations, with most choosing touchscreen. Therefore, voice recognition administrations were not analyzed due to the small sample size. Smartphone SDMT scores showed high agreement with traditional written SDMT administration (CCC = 0.69), with a test–retest reliability estimate provided (*ρ* = 0.87). The measure also displayed correlations with lesion burden (|*ρ*| = 0.45–0.46). Wilcoxon rank-sum tests showed a difference between performance in HVs and PwMS (*p* < 0.0001).

### 6.10. Cognitive Fatigability Assessment Test (cFAST)

Barrios et al. [167] developed a smartphone adaptation of the SDMT with a longer administration (5 min) to assess IPS and cognitive fatigability. The cFAST presents the symbol–digit key at the top of the screen, with a touchscreen digit selection panel at the bottom. One symbol is presented at time; however, if participants do not record a response quickly enough, the item is deemed incorrect, and the test moves on. Symbol–digit pairs are randomized not only with each administration but also with each item, thereby protecting against learning effects within the same administration. Outcomes correlated with the EDSS (|*ρ*| = 0.50–0.60), the Fatigue Scale for Motor and Cognitive Functions (FSMC) (|*ρ*| = 0.38–0.39), and age (|*ρ*| = 0.51–0.66). Change in response time (i.e., changes in item response time across the 5 min administration) was able to classify fatigue (AUC = 0.74).

## 7. Discussion

Six years have passed since the publication of a systematic review of computerized neuropsychological assessment devices applied to PwMS [72]. In just a short time, new technology has encouraged innovation in standardized testing of cognition. In 2019, presentation of test stimuli on tablets was a new development. This platform has become more routine and given way to many smartphone applications. One tablet-based assessment [141] utilizes AI to predict cognitive status after collecting cognitive performance data and other variables. While these developments are innovative and potentially valid, much of the work is preliminary—we as a field must avoid letting the engineering and technology get ahead of the science.

Psychometric science has formed the foundation of cognitive test development as utilized by clinicians and researchers. The traditional path toward acceptance in the field begins with peer-reviewed publications showing adequate test–retest reliability, known-groups validity, and providing normative data. Less critical but often expected are findings demonstrating construct validity and ecological validity (e.g., correlation with brain imaging, employment). Such a validation process was recommended in 2012 for the BICAMS, and there are now 26 country-specific published studies with findings concordant with the published validation standards. Several CNADs have followed similar pathways, showing robust reliability and validity. The Processing Speed Test [78] from the Cleveland Clinic is a classic example. There is a large body of literature that demonstrates its psychometric foundations and utility in clinical research.

The sensitivity and reproducibility of the SDMT in MS was formally recognized in the late 2000s [169,170]. In the late 2000s, studies suggested that SDMT could replace the widely recognized PASAT in the MSFC [66]. Furthermore, the MS Outcomes Assessment Consortium attempted to qualify the SDMT as an approved and accepted outcome measure for MS clinical trials [171]. While the status of the SDMT in this regard remains in question, it has been utilized in a number of phase 3 and phase 4 trials of disease-modifying therapies [70,172,173]. As a result, many test developers have adopted a similar digit–symbol pairing methodology for new digital applications; indeed, one could say that the majority of new CNAD tests reviewed here utilize an SDMT analog. This presents a problem in that we are reaching a point where some MS patients have seen the SDMT or similar tasks many times. There is a risk that the “test,” widely defined, could lose sensitivity in clinical and research settings. The problem could be exacerbated if or when digit–symbol tasks are included in cognitive training interventions. In trial design, one should consider a patient-reported estimate of frequency of exposure to such a test as a covariate. Clinically, these tasks should only be used when necessary to preserve sensitivity. Furthermore, CNADs employing alternative IPS tasks may be beneficial. For example, the Cogstate tasks measure a similar construct(s) but use playing cards as stimuli. This is a unique advantage, and at least one study [112] showed better sensitivity than the traditional SDMT. The NeuroTrax, the NIHTB-CB, and the CANTAB include IPS measures, but with considerably less research on psychometric validity compared to the SDMT. Additionally, there are a few tests that emphasize auditory processing, such as the ATOPS, with promising preliminary findings.

We find that most CNADs still rely on a finger tap or a similar manual response. So as to avoid this confound and to measure IPS independently of motor function, Rao recommended more than 30 years ago that only the oral-response SDMT should be utilized [13,174]. The PST does rely on motor function and correlates with a manual dexterity test within the larger MSPT battery [175]. The CNAD platforms are not very conducive to recording oral responses. Exceptions can be found in elevateMS [153] and Feinstein’s oral-response CNAD SDMT [176,177,178] (reviewed in Wojcik et al. [72] as the auto-SDMT, now named voice-recognition SDMT (VR-SDMT)), where oral responses are captured using voice recognition. Unfortunately, use of this test is limited to their own research setting. We are in the process of validating a voice recognition version of the ATOPS.

Four CNAD-based test batteries have been increasingly utilized in recent years. These include the NeuroTrax, the CBB, the CANTAB, and the NIHTB-CB. Of the four, the former has undergone the most intensive study and has been utilized in a number of research studies examining a wide range of topics, such as longitudinal changes in cognition and correlation with brain imaging. There were 23 studies covered by the Wojick review and 15 that were considered herein.

In 2020 [118], our group published a study showing that technician-administered and self-administered procedures for the PST and the CBB produce similar results. Researchers investigating both tests have since verified the validity of self-administration [101,110]. However, in one CBB study, healthy older adults showed some differences on the DT, the OCLT, and the OBT when performing at home [117]. How well this research applies to routine clinical care requires further study. We find that self-administration is sometimes assumed to be a valid approach without such prospective comparative studies.

There is variability in treating normative data as an academic undertaking, with data subject to peer review, versus treating these data as proprietary for commercial use. This is a new development in clinical neuropsychology. It is not unique to clinical neuropsychology. There are quantitative brain imaging software packages [179] that are available to MRI providers using proprietary normative data processing. The user gets a percentile for a given structure but has no way to judge the validity of the result, whether the underlying distribution is Gaussian, etc. With the rapid expansion of technology that is readily available to entrepreneurs, psychometric test development is likely to continue down the same path.

A strength of the burgeoning research base of the BICAMS is an opportunity to compare findings across languages and countries. Because the outcome measures are identical across studies, there is an opportunity to use linear regression models to normalize data, accounting not only for demographic variables but also country or language as beta weights. Such an approach was taken by Smerbeck et al. [56] in a model including Argentina, Brazil, Czech Republic, Iran, and the United States. There is little research of this nature to be found among the CNADs reviewed here, particularly new measures, albeit with a few exceptions. For example, the CANTAB’s design utilizes visuospatial tasks to minimize the effects of language and culture. It has been studied in Brazilian [180], Chinese [181], German [182], and Singaporean [183] populations, among others. The NIHTB-CB has been translated into multiple languages. Normative data collected from a Spanish-speaking sample were published [184], with correction for demographic variables and experience with the Spanish language. The NIHTB-CB was recently studied in Western Kenya, resulting in normative data derived from translations into Swahili and Dholuo [185]. Other research is ongoing using V3 in other languages. In the case of the CBB, a study from Taiwan showed good known-groups validity comparing mild cognitive impairment to HVs [186]. There is also research on the construct validity of the CBB in other cultural contexts, such as Uganda [187,188]. PST normative data from a Japanese cohort are also available [189]. To our knowledge, known-groups validity research in non-English-speaking MS samples are few, unlike the case of the BICAMS.

Considering this review, what may be the optimal strategy for the longitudinal study of cognitive function in MS patients? First, despite the technological innovations reviewed here, the psychometric foundation, acceptability, widespread use in the field, and validity across cultures and languages of the BICAMS would lead us to recommend its use as a primary outcome for natural history or clinical trial work. We would also recommend that it be used infrequently, at 6-month intervals or longer. That way, the few alternate forms available could be used sparingly. For more frequent evaluations, we would favor a computerized assessment such as the PST or the CBB, as they are less confounded with practice or learning effects. As technology continues to accelerate, future test development may incorporate the use of artificial intelligence and other features such as integration into electronic health records. Furthermore, given the numerous measures that have emerged since 2019, further validation efforts are required for most assessments.

**Figure 1:**
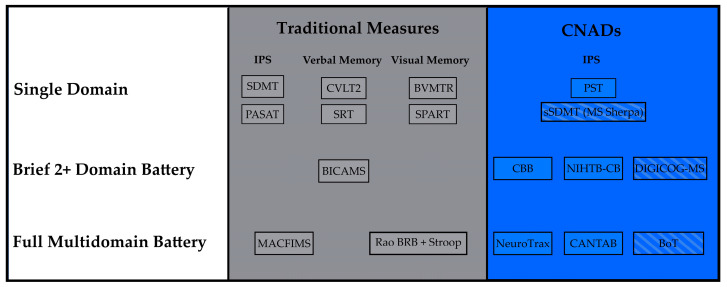
Presented here are conventional tests with robust published data supporting known-groups validity, test–retest reliability, ecological validity, and construct validity. The digital section includes tests that have substantial evidence of reliability and validity. Those with diagonal lines have preliminary findings that suggest known-groups validity and/or test–retest reliability. Note: CNADs = computerized neuropsychological assessment devices; IPS = information processing speed; SDMT = Symbol Digit Modalities Test; PASAT = Paced Auditory Serial Addition Test; CVLT2 = California Verbal Learning Test Second Edition; SRT = Selective Reminding Test; BVMTR = Brief Visuospatial Memory Test—Revised; SPART = 10/36 Spatial Recall Test; BICAMS = Brief International Cognitive Assessment for Multiple Sclerosis; MACFIMS = Minimal Assessment of Cognitive Function in Multiple Sclerosis; BRB = Brief Repeatable Battery; PST = Processing Speed Test; MS = multiple sclerosis; sSDMT = Smartphone-Adapted SDMT; CBB = Cogstate Brief Battery; NIHTB-CB = National Institute of Health Toolbox Cognition Battery; DIGICOG-MS = Digital Assessment of Cognitive Impairment in Multiple Sclerosis; CANTAB = Cambridge Neuropsychological Test Automated Battery; BoT = Brain on Track.

Figure 1 presents the CNADs and traditional tests with the most support in this review. One post-2019 CNAD was added per category, although results are preliminary. These measures are indicated by diagonal lines. Although other SDMT variants show psychometric evidence similar to the sSDMT, they are limited to tablet or computer platforms, whereas the sSDMT runs on smartphones. The DIGICOG-MS shows particular promise due to the high test–retest reliability and concordance with traditional measures, although known-groups validity is yet to be demonstrated. Initial validation of the BoT in PwMS showed strong known-groups validity and test–retest reliability.

This narrative review should be interpreted in light of important limitations. First, this is not a systematic review, nor did it adhere to a predefined search protocol. Therefore, results may be subject to publication and selection biases. Given the expanding scope of CNADs in MS, future research should conduct an updated systematic review on this topic. Second, we reviewed measures from Wojcik et al. [72] only if substantial updates were found in the literature. Some measures reviewed in 2019 may remain relevant, despite limited recent publications. Lastly, many measures aim to be self-administered or to involve only limited involvement of the technician. However, few studies directly examine the influence of technician presence or the validity of unsupervised testing. Differences between supervised and unsupervised testing are likely to vary by individual, as some patients may take unsupervised testing less seriously, while others may put forth a more valid performance once free from the potentially intimidating clinical environment.

## 8. Conclusions

We have presented an updated review of the BICAMS and examined 30 CNAD batteries or tests applied in MS patients. For the BICAMS, in over 26 studies across culture and language (Table 1), the effect size (Cohen’s d) for discriminating patients from HVs is 0.9 for the SDMT and 0.6 for the memory tests. The average test–retest reliabilities are 0.9 and 0.8, respectively. Some of the most widely researched CNAD tests have comparable validity, but the results from newly presented digital platforms should be considered preliminary. We are seeing new frontiers in cognitive testing that were not available just 5 years ago.

## Figures and Tables

**Table 1:** International validation studies of the Brief International Cognitive Assessment for Multiple Sclerosis (BICAMS). Known-groups validity is represented by the effect size (Cohen’s d) of comparisons between PwMS and HVs. Test–retest reliability coefficients in PwMS are reported. For detailed findings, see the systematic review and meta-analysis published by Potticary and Langdon (2023) [21].

Study	Country	Participants		Known-Groups Validity			Test–Retest Reliability **		
		PwMS	HVs	SDMT	CVLT2	BVMTR	SDMT	CVLT2	BVMTR
Alarcón et al. [24] *	Colombia	50	100	0.59	0.38	0.58	0.93	0.89	0.86
Betscher et al. [25]	Poland	61	61	0.77	0.44	0.46	0.90	0.83	0.84
Botchorishvili et al. [26]	Georgia	68	68	0.86	0.74	0.48	0.87	0.83	0.80
Costers et al. [27]	Belgium	97	97	0.76	0.11	0.45	NR	NR	NR
Darwish et al. [28] *	Lebanon	43	180	0.90	0.31	0.30	0.92	0.64	0.83
Drulovic et al. [29]	Serbia	500	69	0.63	0.24	0.53	0.70	0.70	0.70
Dusankova et al. [30]	Czech Republic	367	134	1.24	0.78	0.95	NR	NR	NR
Estiasari et al. [31]	Indonesia	40	66	1.52	0.87	1.10	0.86	0.81	0.83
Evdoshenko et al. [32]	Russia	98	86	0.73	0.30	0.19	0.82	0.85	0.70
Farghaly et al. [33]	Egypt	90	85	0.96	0.62	0.64	0.85	0.61	0.68
Filser et al. [34] *	Germany	172	100	0.74	0.02	0.42	0.85	0.72	0.71
Giedraitiene et al. [35]	Lithuania	50	20	1.13	1.08	1.03	0.91	0.81	0.82
Hämäläinen et al. [36]	Finland	65	45	1.21	0.74	0.73	0.86	0.84	0.71
Marstrand et al. [37]	Denmark	65	65	0.51	0.38	0.45	0.90	0.82	0.68
Maubeuge et al. [38] *	France	123	276	0.88	0.80	0.62	0.89	0.78	0.67
Niino et al. [39]	Japan	156	126	1.07	0.61	0.67	0.93	0.82	0.77
O’Connell et al. [40]	Ireland	67	66	0.85	0.86	0.44	NR	NR	NR
Ozakbas et al. [41]	Turkey	173	153	0.92	0.84	0.63	0.86	0.90	0.87
Polychroniadou et al. [42] *	Greece	44	79	1.10	0.44	0.50	0.96	0.97	0.95
Sandi et al. [43]	Hungary	65	65	0.80	0.38	0.57	0.88	0.74	0.87
Skorve et al. [44]	Norway	65	68	0.36	0.61	0.50	NR	NR	NR
Souissi et al. [45] *	Tunisia	104	104	0.78	0.61	0.50	NR	NR	NR
Sousa et al. [46]	Portugal	105	60	0.64	0.48	0.44	0.90	0.71	0.84
Spedo et al. [47]	Brazil	58	58	0.79	0.97	0.48	0.86	0.84	0.77
Vanotti et al. [48]	Argentina	50	100	0.90	0.89	0.42	0.95	0.87	0.82
Walker et al. [49]	Canada	57	51	0.97	0.67	0.97	0.87	0.74	0.68
Mean (unweighted)		109.0	91.6	0.9	0.6	0.6	0.9	0.8	0.8

Note: PwMS = people with multiple sclerosis; HVs = healthy volunteers; SDMT = Symbol Digit Modalities Test; CVLT2 = California Verbal Learning Test Second Edition; BVMTR = Brief Visuospatial Memory Test—Revised; NR = not reported. * A verbal learning and memory test previously validated in the country of study utilized in lieu of CVLT2. ** Test–retest reliability based on Pearson or intraclass coefficients.

**Table 2:** Methodologies and procedure summary for CNADs.

Test	Stimulus Sensory Modality	Response Modality	Scoring Automated?	Normative Data	Technician Involvement if Applied in Clinic Setting
NeuroTrax [75,84,85,86,87,88,89,90,91,92,93,94,95]	Visual	Manual	Yes	Published norms	Oversight of testing; normalization automated
PST [78,96,97,98,99,100,101,102,103,104,105,106,107,108,109]	Visual	Manual	Yes	Published norms	SA or oversight of testing; normalization automated
CBB [74,110,111,112,113,114,115,116,117,118]	Visual	Manual	Yes	Published norms	SA or oversight of testing; normalization automated
CANTAB [82,119,120,121,122]	Visual	Manual	Yes	Published norms for some tests	SA or oversight of testing; normalization automated
NIHTB-CB [83,123,124]	Visual	Manual/Oral	Yes	Published norms	Administer/supervise tests; normalization automated
ATOPS [125,126]	Auditory	Oral	Yes	HV descriptives in individual studies	Administer test; derive normed values
ACE [127]	Visual	Manual	Yes	Not found	Oversight of testing
EVO Monitor [128,129]	Visual	Manual	Yes	Not found	Oversight of testing
iCognition [81]	Visual	Manual	Yes	Published norms	Oversight of testing; derive normed values
Symbol Search/Dot Memory [130,131,132,133]	Visual	Manual	Yes	HV descriptives in individual studies	Oversight followed by SA; derive normed values
VRAT [134,135]	Visual	Manual	Yes	HV descriptives in individual studies	Oversight of testing; derive normed values
BACH [136,137]	Visual	Manual	Yes	HV descriptives in individual studies	SA; derive normed values
MSReactor [138,139,140]	Visual	Manual	Yes	Not found	SA or oversight of testing
ICA [141,142]	Visual	Manual	Yes	AI used to determine cognitive status	Oversight of testing; normalization automated
BoT [143,144,145]	Auditory/Visual	Manual	Yes	HV descriptives in individual studies	Oversight followed by SA; derive normed values
Multiple Screener [146,147]	Auditory/Visual	Manual	Yes	Published norms	SA; derive normed values
iCAMS [148]	Auditory/Visual	Manual/Oral	Partially	Used traditional BICAMS norms	Administer tests; normalization automated
BCCAMS [149]	Auditory/Visual	Manual/Oral	Partially	Published norms	Administer or supervise tests; derive normed values
iBICAMS [150]	Auditory/Visual	Manual/Oral	Partially	Used traditional BICAMS norms	Administer tests; normalization automated
DIGICOG-MS [151]	Auditory/Visual	Manual/Oral	Partially	Not found	Administer tests
Konectom CPS Test [152]	Visual	Manual	Yes	HV descriptives in individual studies	Oversight followed by SA; derive normed values
elevateMS [153]	Visual	Oral	Yes	Not found	SA
sSDMT (MS Sherpa) [154,155]	Visual	Manual	Yes	HV descriptives in individual studies	SA; derive normed values
Floodlight Open [156,157,158,159,160]	Visual	Manual	Yes	Dataset is publicly available online	SA; derive normed values
eSDMT [161]	Visual	Manual	Yes	Not found	SA
CoRe [162]	Visual	Manual	Yes	HV descriptives in individual studies	Oversight of testing; derive normed values
MCT [163,164]	Visual	Manual	Yes	Not found	Oversight of testing
MD-SDMT [165]	Visual	Manual	Yes	Not found	Oversight of testing
NeuFun SDMT [166]	Visual	Manual	Yes	Dataset is publicly available online	SA or oversight of testing; derive normed values
cFAST [167]	Visual	Manual	Yes	Not found	Oversight of testing

Note: For response modality, “manual” indicates the use of hands to write, draw, or select responses via touchscreen, keyboard, or computer mouse. All oral responses are speech in the form of words or numbers. Scoring refers to the process of converting responses to raw test scores. For normative data, “published norms” indicates normative data are presented in a peer-reviewed article or public domain test manual. “HV descriptives in individual studies” indicates that HV means and standard deviations (or standard error) are included in at least one peer-reviewed publication. “Not found” indicates that HV mean and standard deviation data could not be located. “AI used to determine cognitive status” indicates an artificial intelligence model was used to determine cognitive status. “Used traditional BICAMS norms” indicates traditional paper-based BICAMS norms were used. “Dataset is publicly available online” indicates a study dataset, including HV data, is publicly available. For “technician involvement if applied in clinic setting,” any technician involvement during assessment is described. SA indicates the test may be self-administered without technician oversight. HV = healthy volunteer; SA = self-administered; AI = artificial intelligence; PST = Processing Speed Test; CBB = Cogstate Brief Battery; CANTAB = Cambridge Neuropsychological Test Automated Battery; NIHTB-CB = National Institute of Health Toolbox Cognition Battery; ATOPS = Auditory Test of Processing Speed; ACE = Adaptive Cognitive Evaluation; VRAT = Virtual Reality Attention Tracker; BACH = Brief Assessment of Cognitive Health; ICA = Integrated Cognitive Assessment; BoT = Brain on Track; BCCAMS = Brief Computerized Cognitive Assessment for Multiple Sclerosis; DIGICOG-MS = Digital Assessment of Cognitive Impairment in Multiple Sclerosis; CPS = Cognitive Processing Speed; sSDMT = Smartphone-Adapted Symbol Digit Modalities Test; eSDMT = Electronic Symbol Digit Modalities Test; CoRe = Cognition Reaction; MCT = Mobile Cognition Test; MD-SDMT = Modified Symbol Digit Modalities Test; cFAST = Cognitive Fatigability Assessment Test.

## References

[1] Filippi M., Bar-Or A., Piehl F., Preziosa P., Solari A., Vukusic S., Rocca M.A. (2018). Multiple sclerosis. Nat. Rev. Dis. Primers.

[2] Amato M., Hakiki B., Goretti B., Rossi F., Stromillo M.L., Giorgio A., Roscio M., Ghezzi A., Guidi L., Bartolozzi M. (2012). Association of MRI metrics and cognitive impairment in radiologically isolated syndromes. Neurology.

[3] Benedict R.H., DeLuca J., Enzinger C., Geurts J.J., Krupp L.B., Rao S.M. (2017). Neuropsychology of multiple sclerosis: Looking back and moving forward. J. Int. Neuropsychol. Soc..

[4] Ruano L., Portaccio E., Goretti B., Niccolai C., Severo M., Patti F., Cilia S., Gallo P., Grossi P., Ghezzi A. (2017). Age and disability drive cognitive impairment in multiple sclerosis across disease subtypes. Mult. Scler. J..

[5] De Meo E., Portaccio E., Giorgio A., Ruano L., Goretti B., Niccolai C., Patti F., Chisari C.G., Gallo P., Grossi P. (2021). Identifying the distinct cognitive phenotypes in multiple sclerosis. JAMA Neurol..

[6] Vitturi B.K., Rahmani A., Dini G., Montecucco A., Debarbieri N., Sbragia E., Bandiera P., Ponzio M., Battaglia M.A., Manacorda T. (2022). Occupational outcomes of people with multiple sclerosis: A scoping review. BMJ Open.

[7] Benedict R.H., Rodgers J.D., Emmert N., Kininger R., Weinstock-Guttman B. (2014). Negative work events and accommodations in employed multiple sclerosis patients. Mult. Scler. J..

[8] Marafioti G., Cardile D., Culicetto L., Quartarone A., Lo Buono V. (2024). The impact of social cognition deficits on quality of life in multiple sclerosis: A scoping review. Brain Sci..

[9] Gómez-Melero S., Caballero-Villarraso J., Escribano B.M., Galvao-Carmona A., Túnez I., Agüera-Morales E. (2024). Impact of Cognitive Impairment on Quality of Life in Multiple Sclerosis Patients—A Comprehensive Review. J. Clin. Med..

[10] Hakim E.A., Bakheit A., Bryant T., Roberts M., McIntosh-Michaelis S., Spackman A., Martin J., McLellan D. (2000). The social impact of multiple sclerosis-a study of 305 patients and their relatives. Disabil. Rehabil..

[11] Benedict R.H., Cox D., Thompson L.L., Foley F., Weinstock-Guttman B., Munschauer F. (2004). Reliable screening for neuropsychological impairment in multiple sclerosis. Mult. Scler. J..

[12] Macaron G., Farah N., Charbonneau S., Morrow S.A., Zertal A., Saint-Amour D., Duquette P., Rouleau I. (2025). Addressing patient-reported cognitive impairment in multiple sclerosis clinical practice: A challenging endeavor. Mult. Scler. J..

[13] Rao S.M. (1990). A Manual for the Brief Repeatable Battery of Neuropsychological Tests in Multiple Sclerosis.

[14] Benedict R.H., Fischer J.S., Archibald C.J., Arnett P.A., Beatty W.W., Bobholz J., Chelune G.J., Fisk J.D., Langdon D.W., Caruso L. (2002). Minimal neuropsychological assessment of MS patients: A consensus approach. Clin. Neuropsychol..

[15] Benedict R.H., Cookfair D., Gavett R., Gunther M., Munschauer F., Garg N., Weinstock-Guttman B. (2006). Validity of the minimal assessment of cognitive function in multiple sclerosis (MACFIMS). J. Int. Neuropsychol. Soc..

[16] Langdon D., Amato M., Boringa J., Brochet B., Foley F., Fredrikson S., Hämäläinen P., Hartung H., Krupp L., Penner I. (2012). Recommendations for a brief international cognitive assessment for multiple sclerosis (BICAMS). Mult. Scler. J..

[17] Smith A. (1982). Symbol Digit Modalities Test: Manual.

[18] Delis D.C., Kramer J.H., Kaplan E., Ober B.A. (2000). California verbal learning test. Assessment.

[19] Benedict R.H. (1997). Brief visuospatial memory test--revised. Psychol. Assess. Resour..

[20] Benedict R.H., Amato M.P., Boringa J., Brochet B., Foley F., Fredrikson S., Hamalainen P., Hartung H., Krupp L., Penner I. (2012). Brief International Cognitive Assessment for MS (BICAMS): International standards for validation. BMC Neurol..

[21] Potticary H., Langdon D. (2023). A systematic review and meta-analysis of the brief cognitive assessment for multiple sclerosis (BICAMS) international validations. J. Clin. Med..

[22] Spiezia A.L., Pontillo G., Falco F., Eliano M., Lamagna F., Esposito A., Di Monaco C., Nicolella V., Novarella F., Moccia M. (2025). Identifying neuropsychological phenotypes in multiple sclerosis using latent profile analysis. Eur. J. Neurol..

[23] Batista S., Zivadinov R., Hoogs M., Bergsland N., Heininen-Brown M., Dwyer M.G., Weinstock-Guttman B., Benedict R.H. (2012). Basal ganglia, thalamus and neocortical atrophy predicting slowed cognitive processing in multiple sclerosis. J. Neurol..

[24] Alarcón A.N., Ayala O.D., García J.R., Montañés P. (2020). Validation of the Brief International Cognitive Assessment for Multiple Sclerosis (BICAMS) in a Colombian Population. Mult. Scler. Relat. Disord..

[25] Betscher E., Guenter W., Langdon D.W., Bonek R. (2021). Polish validation of the Brief International Cognitive Assessment for Multiple Sclerosis (BICAMS battery): Correlation of cognitive impairment with mood disorders and fatigue. Neurol. I Neurochir. Pol..

[26] Botchorishvili N., Shiukashvili N., Mikeladze N., Dzagnidze A., Mikava N., Tighashvili M., Janelidze M. (2021). Validity and reliability of the Georgian-language brief international cognitive assessment for multiple sclerosis (BICAMS). BMC Neurol..

[27] Costers L., Gielen J., Eelen P.L., Schependom J.V., Laton J., Remoortel A.V., Vanzeir E., Wijmeersch B.V., Seeldrayers P., Haelewyck M.C. (2017). Does including the full CVLT-II and BVMT-R improve BICAMS? Evidence from a Belgian (Dutch) validation study. Mult. Scler. Relat. Disord..

[28] Darwish H., Zeinoun P., Farran N., Ghusn H., Yamout B., Khoury S.J. (2022). The Brief International Cognitive Assessment in Multiple Sclerosis (BICAMS): Validation in Arabic and Lebanese Normative Values. J. Int. Neuropsychol. Soc..

[29] Drulović J., Tončev G., Nadj Č., Obradović D., Eraković J., Mesaroš Š., Čukić M., Aleksić D., Andabaka M., Ivanović J. (2022). Validation of the Brief International Cognitive Assessment for Multiple Sclerosis (BICAMS) in a large cohort of relapsing-remitting MS patients. Acta Clin. Croat..

[30] Dusankova J.B., Kalincik T., Havrdova E., Benedict R.H. (2012). Cross cultural validation of the Minimal Assessment of Cognitive Function in Multiple Sclerosis (MACFIMS) and the Brief International Cognitive Assessment for Multiple Sclerosis (BICAMS). Clin. Neuropsychol..

[31] Estiasari R., Fajrina Y., Lastri D.N., Melani S., Maharani K., Imran D., Pangeran D., Sitorus F. (2019). Validity and Reliability of Brief International Cognitive Assessment for Multiple Sclerosis (BICAMS) in Indonesia and the Correlation with Quality of Life. Neurol. Res. Int..

[32] Evdoshenko E., Laskova K., Shumilina M., Nekrashevich E., Andreeva M., Neofidov N., Kalinin I., Nikitchenko D., Rogozina A., Kupaeva A. (2022). Validation of the Brief International Cognitive Assessment for Multiple Sclerosis (BICAMS) in the Russian Population. J. Int. Neuropsychol. Soc..

[33] Farghaly M., Langdon D.W., Shalaby N.M., Shehata H.S., Abokrysha N.T., Hassan A., Hegazy M.I., Elmazny A., Ahmed S., Shaheen S. (2021). Reliability and validity of Arabic version of the brief international cognitive assessment for multiple sclerosis: Egyptian dialect. Egypt. J. Neurol. Psychiatry Neurosurg..

[34] Filser M., Schreiber H., Pöttgen J., Ullrich S., Lang M., Penner I.K. (2018). The Brief International Cognitive Assessment in Multiple Sclerosis (BICAMS): Results from the German validation study. J. Neurol..

[35] Giedraitienė N., Kizlaitienė R., Kaubrys G. (2015). The BICAMS Battery for Assessment of Lithuanian-Speaking Multiple Sclerosis Patients: Relationship with Age, Education, Disease Disability, and Duration. Med. Sci. Monit..

[36] Hämäläinen P., Leo V., Therman S., Ruutiainen J. (2021). Validation of the Finnish version of the Brief International Cognitive Assessment for Multiple Sclerosis (BICAMS) and evaluation of the applicability of the Multiple Sclerosis Neuropsychological Questionnaire (MSNQ) and the Fatigue Scale for Motor and Cognitive Functions (FSMC). Brain Behav..

[37] Marstrand L., Østerberg O., Walsted T., Skov A.C., Schreiber K.I., Sellebjerg F. (2020). Brief international cognitive assessment for multiple sclerosis (BICAMS): A danish validation study of sensitivity in early stages of MS. Mult. Scler. Relat. Disord..

[38] Maubeuge N., Deloire M.S.A., Brochet B., Erhlé N., Charré-Morin J., Saubusse A., Ruet A. (2021). French validation of the Brief International Cognitive Assessment for Multiple Sclerosis. Rev. Neurol..

[39] Niino M., Fukazawa T., Kira J.I., Okuno T., Mori M., Sanjo N., Ohashi T., Fukaura H., Fujimori J., Shimizu Y. (2017). Validation of the Brief International Cognitive Assessment for Multiple Sclerosis in Japan. Mult. Scler. J. Exp. Transl. Clin..

[40] O’Connell K., Langdon D., Tubridy N., Hutchinson M., McGuigan C. (2015). A preliminary validation of the brief international cognitive assessment for multiple sclerosis (BICAMS) tool in an Irish population with multiple sclerosis (MS). Mult. Scler. Relat. Disord..

[41] Ozakbas S., Yigit P., Cinar B.P., Limoncu H., Kahraman T., Kösehasanoğulları G. (2017). The Turkish validation of the Brief International Cognitive Assessment for Multiple Sclerosis (BICAMS) battery. BMC Neurol..

[42] Polychroniadou E., Bakirtzis C., Langdon D., Lagoudaki R., Kesidou E., Theotokis P., Tsalikakis D., Poulatsidou K., Kyriazis O., Boziki M. (2016). Validation of the Brief International Cognitive Assessment for Multiple Sclerosis (BICAMS) in Greek population with multiple sclerosis. Mult. Scler. Relat. Disord..

[43] Sandi D., Rudisch T., Füvesi J., Fricska-Nagy Z., Huszka H., Biernacki T., Langdon D.W., Langane É., Vécsei L., Bencsik K. (2015). The Hungarian validation of the Brief International Cognitive Assessment for Multiple Sclerosis (BICAMS) battery and the correlation of cognitive impairment with fatigue and quality of life. Mult. Scler. Relat. Disord..

[44] Skorve E., Lundervold A.J., Torkildsen Ø., Myhr K.M. (2019). The Norwegian translation of the brief international cognitive assessment for multiple sclerosis (BICAMS). Mult. Scler. Relat. Disord..

[45] Souissi A., Mrabet S., Ferchichi W., Gharbi A., Nasri A., Djebara M.B., Kacem I., Gouider R. (2022). Tunisian version of the brief international cognitive assessment for multiple sclerosis: Validation and normative values. Mult. Scler. Relat. Disord..

[46] Sousa C., Rigueiro-Neves M., Miranda T., Alegria P., Vale J., Passos A.M., Langdon D., Sá M.J. (2018). Validation of the brief international cognitive assessment for multiple sclerosis (BICAMS) in the Portuguese population with multiple sclerosis. BMC Neurol..

[47] Spedo C.T., Frndak S.E., Marques V.D., Foss M.P., Pereira D.A., Carvalho Lde F., Guerreiro C.T., Conde R.M., Fusco T., Pereira A.J. (2015). Cross-cultural Adaptation, Reliability, and Validity of the BICAMS in Brazil. Clin. Neuropsychol..

[48] Vanotti S., Smerbeck A., Benedict R.H., Caceres F. (2016). A new assessment tool for patients with multiple sclerosis from Spanish-speaking countries: Validation of the Brief International Cognitive Assessment for MS (BICAMS) in Argentina. Clin. Neuropsychol..

[49] Walker L.A., Osman L., Berard J.A., Rees L.M., Freedman M.S., MacLean H., Cousineau D. (2016). Brief International Cognitive Assessment for Multiple Sclerosis (BICAMS): Canadian contribution to the international validation project. J. Neurol. Sci..

[50] Goretti B., Niccolai C., Hakiki B., Sturchio A., Falautano M., Minacapelli E., Martinelli V., Incerti C., Nocentini U., Murgia M. (2014). The brief international cognitive assessment for multiple sclerosis (BICAMS): Normative values with gender, age and education corrections in the Italian population. BMC Neurol..

[51] Falco F., Lamagna F., Eliano M., di Monaco C., Trojano L., Lus G., Moccia M., Lauro F., Liccardo T., Chiodi A. (2025). Normative values of the brief international cognitive assessment for multiple sclerosis (BICAMS) in an Italian young adolescent population: The influence of age, sex, and education. Neurol. Sci..

[52] Alboudi A., Hadid A., Ali A.R., Alshaikh F., Aqleh H. (2020). Normative values of the Brief International Cognitive Assessment for Multiple Sclerosis (BICAMS) in an Arab population: Corrected for age, sex and education. Mult. Scler. Relat. Disord..

[53] Spedo C.T., de Assis Pereira D., Frndak S.E., Marques V.D., Barreira A.A., Smerbeck A., da Silva P.H.R., Benedict R.H. (2022). Brief International Cognitive Assessment for Multiple Sclerosis (BICAMS): Discrete and regression-based norms for the Brazilian context. Arq. Neuro-Psiquiatr..

[54] Batum M., Sarıtaş A.Ş., Erdoğan B., Çelebi N., Ak A.K., Mavioğlu H. (2025). The Brief International Cognitive Assessment for Multiple Sclerosis (BICAMS): Normative values with gender, age and education corrections in the Turkish population. Mult. Scler. Relat. Disord..

[55] Penner I., Baijot J., Filser M., Bätge S., Raithel L., Toth E., Renner A., Nagels G. (2025). The Brief International Cognitive Assessment in Multiple Sclerosis (BICAMS): Regression-based norms for German-speaking countries. Eur. J. Neurol..

[56] Smerbeck A., Benedict R.H., Eshaghi A., Vanotti S., Spedo C., Blahova Dusankova J., Sahraian M.A., Marques V.D., Langdon D. (2018). Influence of nationality on the brief international cognitive assessment for multiple sclerosis (BICAMS). Clin. Neuropsychol..

[57] Feinstein A., Amato M.P., Brichetto G., Chataway J., Chiaravalloti N.D., Cutter G., Dalgas U., DeLuca J., Farrell R., Feys P. (2023). Cognitive rehabilitation and aerobic exercise for cognitive impairment in people with progressive multiple sclerosis (CogEx): A randomised, blinded, sham-controlled trial. Lancet Neurol..

[58] American Academy of Neurology (2014). Multiple Sclerosis Quality Measurement Set. https://www.aan.com/siteassets/home-page/policy-and-guidelines/quality/quality-measures/14msmeasureset_pg.pdf.

[59] Sarnataro A., Cuomo N., Russo C.V., Carotenuto A., Lanzillo R., Moccia M., Petracca M., Morra V.B., Saccà F. (2024). Integration of the expanded disability status scale with ambulation, visual and cognitive tests. Neurol. Sci..

[60] Pyle W.H. (1913). The Examination of School Children: A Manual of Directions and Norms.

[61] Whipple G.M. (1910). Manual of Mental and Physical Tests.

[62] Wechsler D. (1939). Wechsler-Bellevue Intelligence Scale.

[63] Wechsler D. (1981). WAIS-R Manual.

[64] Benedict R.H., Weinstock-Guttman B., Fishman I., Sharma J., Tjoa C.W., Bakshi R. (2004). Prediction of neuropsychological impairment in multiple sclerosis: Comparison of conventional magnetic resonance imaging measures of atrophy and lesion burden. Arch. Neurol..

[65] Drake A., Weinstock-Guttman B., Morrow S., Hojnacki D., Munschauer F., Benedict R. (2010). Psychometrics and normative data for the Multiple Sclerosis Functional Composite: Replacing the PASAT with the Symbol Digit Modalities Test. Mult. Scler. J..

[66] Brochet B., Deloire M., Bonnet M., Salort-Campana E., Ouallet J., Petry K., Dousset V. (2008). Should SDMT substitute for PASAT in MSFC? A 5-year longitudinal study. Mult. Scler. J..

[67] Benedict R.H., Morrow S., Rodgers J., Hojnacki D., Bucello M.A., Zivadinov R., Weinstock-Guttman B. (2014). Characterizing cognitive function during relapse in multiple sclerosis. Mult. Scler. J..

[68] McKay K.A., Bedri S.K., Manouchehrinia A., Stawiarz L., Olsson T., Hillert J., Fink K. (2022). Reduction in cognitive processing speed surrounding multiple sclerosis relapse. Ann. Neurol..

[69] Deluca J., Huang D., Cohen J., Cree B.A., Chen Y., Campagnolo D., Harvey D., Sheffield J.K., Comi G., Kappos L. (2019). Assessment of Cognitive Processing Speed in the Phase 3 SUNBEAM Trial Demonstrates Sustained Improvement in Ozanimod-Treated Patients. Americas Committee for Treatment and Research in Multiple Sclerosis (ACTRIMS).

[70] Benedict R.H.B., Tomic D., Cree B.A., Fox R., Giovannoni G., Bar-Or A., Gold R., Vermersch P., Pohlmann H., Wright I. (2021). Siponimod and Cognition in Secondary Progressive Multiple Sclerosis: EXPAND Secondary Analyses. Neurology.

[71] Kane R.L., Kay G.G. (1992). Computerized assessment in neuropsychology: A review of tests and test batteries. Neuropsychol. Rev..

[72] Wojcik C.M., Beier M., Costello K., DeLuca J., Feinstein A., Goverover Y., Gudesblatt M., Jaworski M., Kalb R., Kostich L. (2019). Computerized neuropsychological assessment devices in multiple sclerosis: A systematic review. Mult. Scler. J..

[73] Edgar C., Jongen P.J., Sanders E., Sindic C., Goffette S., Dupuis M., Jacquerye P., Guillaume D., Reznik R., Wesnes K. (2011). Cognitive performance in relapsing remitting multiple sclerosis: A longitudinal study in daily practice using a brief computerized cognitive battery. BMC Neurol..

[74] Darby D., Maruff P., Collie A., McStephen M. (2002). Mild cognitive impairment can be detected by multiple assessments in a single day. Neurology.

[75] Achiron A., Doniger G.M., Harel Y., Appleboim-Gavish N., Lavie M., Simon E.S. (2007). Prolonged response times characterize cognitive performance in multiple sclerosis. Eur. J. Neurol..

[76] Gualtieri C.T., Johnson L.G. (2006). Reliability and validity of a computerized neurocognitive test battery, CNS Vital Signs. Arch. Clin. Neuropsychol..

[77] Akbar N., Honarmand K., Kou N., Feinstein A. (2011). Validity of a computerized version of the symbol digit modalities test in multiple sclerosis. J. Neurol..

[78] Rao S.M., Losinski G., Mourany L., Schindler D., Mamone B., Reece C., Kemeny D., Narayanan S., Miller D.M., Bethoux F. (2017). Processing speed test: Validation of a self-administered, iPad^®^-based tool for screening cognitive dysfunction in a clinic setting. Mult. Scler. J..

[79] Ruet A., Deloire M.S., Charré-Morin J., Hamel D., Brochet B. (2013). A new computerised cognitive test for the detection of information processing speed impairment in multiple sclerosis. Mult. Scler. J..

[80] Foong Y.C., Bridge F., Merlo D., Gresle M., Zhu C., Buzzard K., Butzkueven H., van der Walt A. (2023). Smartphone monitoring of cognition in people with multiple sclerosis: A systematic review. Mult. Scler. Relat. Disord..

[81] Denissen S., Van Laethem D., Baijot J., Costers L., Descamps A., Van Remoortel A., Van Merhaegen-Wieleman A., D’Hooghe M., D’Haeseleer M., Smeets D. (2025). A New Smartphone-Based Cognitive Screening Battery for Multiple Sclerosis (icognition): Validation Study. J. Med. Internet Res..

[82] Lowe C., Rabbitt P. (1998). Test\re-test reliability of the CANTAB and ISPOCD neuropsychological batteries: Theoretical and practical issues. Neuropsychologia.

[83] Zelazo P.D., Anderson J.E., Richler J., Wallner-Allen K., Beaumont J.L., Conway K.P., Gershon R., Weintraub S. (2014). NIH Toolbox Cognition Battery (CB): Validation of executive function measures in adults. J. Int. Neuropsychol. Soc..

[84] Bergmann C., Becker S., Watts A., Sullivan C., Wilken J., Golan D., Zarif M., Bumstead B., Buhse M., Kaczmarek O. (2023). Multiple sclerosis and quality of life: The role of cognitive impairment on quality of life in people with multiple sclerosis. Mult. Scler. Relat. Disord..

[85] Bogaardt H., Golan D., Barrera M.A., Attrill S., Kaczmarek O., Zarif M., Bumstead B., Buhse M., Wilken J., Doniger G.M. (2023). Cognitive impairment, fatigue and depression in multiple sclerosis: Is there a difference between benign and non-benign MS?. Mult. Scler. Relat. Disord..

[86] Covey T.J., Golan D., Doniger G.M., Sergott R., Zarif M., Bumstead B., Buhse M., Kaczmarek O., Mebrahtu S., Bergmann C. (2022). Longitudinal assessment of the relationship between visual evoked potentials and cognitive performance in multiple sclerosis. Clin. Neurophysiol..

[87] Covey T.J., Golan D., Sergott R., Wilken J., Zarif M., Bumstead B., Buhse M., Kaczmarek O., Doniger G.M., Penner I.-K. (2024). Peering further into the mind’s eye: Combining visual evoked potential and optical coherence tomography measures enhances insight into the variance in cognitive functioning in multiple sclerosis. J. Neurol..

[88] Dreyer-Alster S., Gal A., Achiron A. (2022). Optical Coherence Tomography Is Associated with Cognitive Impairment in Multiple Sclerosis. J. Neuro-Ophthalmol..

[89] Dreyer-Alster S., Menascu S., Aloni R., Givon U., Dolev M., Achiron A., Kalron A. (2022). Motoric cognitive risk syndrome in people with multiple sclerosis: Prevalence and correlations with disease-related factors. Ther. Adv. Neurol. Disord..

[90] Glen M., Doniger P. (2014). Guide to Normative Data. https://portal.neurotrax.com/docs/norms_guide.pdf.

[91] Golan D., Doniger G.M., Srinivasan J., Sima D.M., Zarif M., Bumstead B., Buhse M., Van Hecke W., Wilken J., Gudesblatt M. (2020). The association between MRI brain volumes and computerized cognitive scores of people with multiple sclerosis. Brain Cogn..

[92] Golan D., Wilken J., Doniger G.M., Fratto T., Kane R., Srinivasan J., Zarif M., Bumstead B., Buhse M., Fafard L. (2019). Validity of a multi-domain computerized cognitive assessment battery for patients with multiple sclerosis. Mult. Scler. Relat. Disord..

[93] Jackson D.A., Nicholson R., Bergmann C., Wilken J., Kaczmarek O., Bumstead B., Buhse M., Zarif M., Penner I.K., Hancock L.M. (2023). Cognitive impairment in people with multiple sclerosis: Perception vs. performance—Factors that drive perception of impairment differ for patients and clinicians. Mult. Scler. Relat. Disord..

[94] Leach J.M., Cutter G., Golan D., Doniger G., Zarif M., Bumstead B., Buhse M., Kaczmarek O., Sethi A., Covey T. (2022). Measuring cognitive function by the SDMT across functional domains: Useful but not sufficient. Mult. Scler. Relat. Disord..

[95] Zanotto T., Pradeep Kumar D., Golan D., Wilken J., Doniger G.M., Zarif M., Bumstead B., Buhse M., Weller J., Morrow S.A. (2025). Does cognitive performance explain the gap between physiological and perceived fall-risk in people with multiple sclerosis?. Mult. Scler. Relat. Disord..

[96] Aboseif A., Amin M., Bena J., Nakamura K., Macaron G., Ontaneda D. (2024). Association Between Disease-Modifying Therapy and Information Processing Speed in Multiple Sclerosis. Int. J. MS Care.

[97] Chan C.K., Tian F., Pimentel Maldonado D., Mowry E.M., Fitzgerald K.C. (2021). Depression in multiple sclerosis across the adult lifespan. Mult. Scler. J..

[98] Conway D.S., Bermel R.A., Planchon S.M. (2022). The relationship between cognition, education, and employment in multiple sclerosis patients. Mult. Scler. J. Exp. Transl. Clin..

[99] Foong Y.C., Merlo D., Gresle M., Zhu C., Buzzard K., Lechner-Scott J., Barnett M., Wang C., Taylor B.V., Kalincik T. (2025). Longitudinal Trajectories of Digital Cognitive Biomarkers for Multiple Sclerosis. Ann. Clin. Transl. Neurol..

[100] Galioto R., Macaron G., Lace J.W., Ontaneda D., Rao S.M. (2021). Is computerized screening for processing speed impairment sufficient for identifying MS-related cognitive impairment in a clinical setting?. Mult. Scler. Relat. Disord..

[101] Hechenberger S., Helmlinger B., Tinauer C., Jauk E., Ropele S., Heschl B., Wurth S., Damulina A., Eppinger S., Demjaha R. (2024). Evaluation of a self-administered iPad(^®^)-based processing speed assessment for people with multiple sclerosis in a clinical routine setting. J. Neurol..

[102] Jaworski M.G., Gillies J.K., Youngs M., Wojcik C., Santivasci C., Jakimovski D., Bergsland N., Weinstock-Guttman B., Benedict R.H. (2023). Predicting employment deterioration with the Processing Speed Test (PST) and SDMT in multiple sclerosis. Mult. Scler. J..

[103] Labiano-Fontcuberta A., Costa-Frossard L., Sainz de la Maza S., Rodríguez-Jorge F., Chico-García J.L., González P.N., Monreal E. (2023). Predictive models of multiple sclerosis-related cognitive performance using routine clinical practice predictors. Mult. Scler. Relat. Disord..

[104] Labiano-Fontcuberta A., Costa-Frossard L., Sainz de la Maza S., Rodríguez-Jorge F., Chico-García J.L., Monreal E. (2022). The effect of timing of high-efficacy therapy on processing speed performance in multiple sclerosis. Mult. Scler. Relat. Disord..

[105] Macaron G., Baldassari L.E., Nakamura K., Rao S.M., McGinley M.P., Moss B.P., Li H., Miller D.M., Jones S.E., Bermel R.A. (2020). Cognitive processing speed in multiple sclerosis clinical practice: Association with patient-reported outcomes, employment and magnetic resonance imaging metrics. Eur. J. Neurol..

[106] Rao S.M., Galioto R., Sokolowski M., McGinley M., Freiburger J., Weber M., Dey T., Mourany L., Schindler D., Reece C. (2020). Multiple Sclerosis Performance Test: Validation of self-administered neuroperformance modules. Eur. J. Neurol..

[107] Rao S.M., Sokolowski M., Strober L.B., Miller J.B., Norman M.A., Levitt N., Williams J.R., de Moor C. (2022). Multiple sclerosis performance test (MSPT): Normative study of 428 healthy participants ages 18 to 89. Mult. Scler. Relat. Disord..

[108] Rhodes J.K., Schindler D., Rao S.M., Venegas F., Bruzik E.T., Gabel W., Williams J.R., Phillips G.A., Mullen C.C., Freiburger J.L. (2019). Multiple Sclerosis Performance Test: Technical Development and Usability. Adv. Ther..

[109] Rudick R.A., Miller D., Bethoux F., Rao S.M., Lee J.-C., Stough D., Reece C., Schindler D., Mamone B., Alberts J. (2014). The Multiple Sclerosis Performance Test (MSPT): An iPad-based disability assessment tool. J. Vis. Exp. JoVE.

[110] Banh T., Jin C., Neuhaus J., Mackin R.S., Maruff P., Stricker N., Weiner M.W., Nosheny R.L. (2022). Unsupervised Performance of the CogState Brief Battery in the Brain Health Registry: Implications for Detecting Cognitive Decline. J. Prev. Alzheimer’s Dis..

[111] Cho H., Pilloni G., Tahsin R., Best P., Krupp L., Oh C., Charvet L. (2023). Moving intra-individual variability (IIV) towards clinical utility: IIV measured using a commercial testing platform. J. Neurol. Sci..

[112] Eilam-Stock T., Shaw M.T., Krupp L.B., Charvet L.E. (2021). Early neuropsychological markers of cognitive involvement in multiple sclerosis. J. Neurol. Sci..

[113] Govindarajan S.T., Liu Y., Parra Corral M.A., Bangiyev L., Krupp L., Charvet L., Duong T.Q. (2021). White matter correlates of slowed information processing speed in unimpaired multiple sclerosis patients with young age onset. Brain Imaging Behav..

[114] Kalinowska-Lyszczarz A., Tillema J.M., Tobin W.O., Guo Y., Weigand S.D., Metz I., Brück W., Lassmann H., Giraldo-Chica M., Port J.D. (2023). Long-term clinical, imaging and cognitive outcomes association with MS immunopathology. Ann. Clin. Transl. Neurol..

[115] Krupp L.B., Waubant E., Waltz M., Casper T.C., Belman A., Wheeler Y., Ness J., Graves J., Gorman M., Benson L. (2023). A new look at cognitive functioning in pediatric MS. Mult. Scler. J..

[116] Pilloni G., Casper T.C., Mar S., Ness J., Schreiner T., Waltz M., Waubant E., Weinstock-Guttman B., Wheeler Y., Krupp L. (2024). Increased intraindividual variability (IIV) in reaction time is the earliest indicator of cognitive change in MS: A two-year observational study. Int. J. Clin. Health Psychol..

[117] Stricker N.H., Lundt E.S., Alden E.C., Albertson S.M., Machulda M.M., Kremers W.K., Knopman D.S., Petersen R.C., Mielke M.M. (2020). Longitudinal Comparison of in Clinic and at Home Administration of the Cogstate Brief Battery and Demonstrated Practice Effects in the Mayo Clinic Study of Aging. J. Prev. Alzheimer’s Dis..

[118] Wojcik C.M., Rao S.M., Schembri A.J., Drake A.S., Maruff P., Schindler D., Alberts J., Yasin F., Pol J., Weinstock-Guttman B. (2020). Necessity of technicians for computerized neuropsychological assessment devices in multiple sclerosis. Mult. Scler. J..

[119] Giedraitiene N., Kaubrys G. (2019). Distinctive Pattern of Cognitive Disorders During Multiple Sclerosis Relapse and Recovery Based on Computerized CANTAB Tests. Front. Neurol..

[120] Karlsen R.H., Karr J.E., Saksvik S.B., Lundervold A.J., Hjemdal O., Olsen A., Iverson G.L., Skandsen T. (2022). Examining 3-month test-retest reliability and reliable change using the Cambridge Neuropsychological Test Automated Battery. Appl. Neuropsychol. Adult.

[121] Lenehan M.E., Summers M.J., Saunders N.L., Summers J.J., Vickers J.C. (2016). Does the Cambridge Automated Neuropsychological Test Battery (CANTAB) Distinguish Between Cognitive Domains in Healthy Older Adults?. Assessment.

[122] Talebi M., Majdi A., Kamari F., Sadigh-Eteghad S. (2020). The Cambridge Neuropsychological Test Automated Battery (CANTAB) Versus the Minimal Assessment of Cognitive Function in Multiple Sclerosis (MACFIMS) for the Assessment of Cognitive Function in Patients with Multiple Sclerosis. Mult. Scler. Relat. Disord..

[123] LaForte E.M., Hook J.N., Giella A.K. (2024). National Institutes of Health (NIH) Toolbox® V3 Technical Manual.

[124] Manglani H.R., Fisher M.E., Duraney E.J., Nicholas J.A., Prakash R.S. (2022). A promising cognitive screener in multiple sclerosis: The NIH toolbox cognition battery concords with gold standard neuropsychological measures. Mult. Scler. J..

[125] Jakimovski D., Zivadinov R., Weinstock Z., Burnham A., Wicks T.R., Suchan C., Sciortino T., Schweser F., Bergsland N., Dwyer M.G. (2024). Cognitive function in severe progressive multiple sclerosis. Brain Commun..

[126] Weinstock Z.L., Jaworski M., Dwyer M.G., Jakimovski D., Burnham A., Wicks T.R., Youngs M., Santivasci C., Cruz S., Gillies J. (2023). Auditory Test of Processing Speed: Preliminary validation of a smartphone-based test of mental speed. Mult. Scler. J..

[127] Hsu W.-Y., Rowles W., Anguera J.A., Anderson A., Younger J.W., Friedman S., Gazzaley A., Bove R. (2021). Assessing cognitive function in multiple sclerosis with digital tools: Observational study. J. Med. Internet Res..

[128] Hsu W.Y., Rowles W., Anguera J.A., Zhao C., Anderson A., Alexander A., Sacco S., Henry R., Gazzaley A., Bove R. (2021). Application of an Adaptive, Digital, Game-Based Approach for Cognitive Assessment in Multiple Sclerosis: Observational Study. J. Med. Internet Res..

[129] Nylander A., Anderson A., Rowles W., Hsu S., Lazar A.A., Mayoral S.R., Pease-Raissi S.E., Green A., Bove R. (2023). Re-WRAP (Remyelination for women at risk of axonal loss and progression): A phase II randomized placebo-controlled delayed-start trial of Bazedoxifene for myelin repair in multiple sclerosis. Contemp. Clin. Trials.

[130] Goga J.J., Ginell K.M., Ng Y.T., Ehde D.M., Alschuler K.N., Sliwinski M.J., Fritz N.E., Kratz A.L. (2025). Feasibility, reliability, and validity of ambulatory smartphone-administered cognitive tests in multiple sclerosis. Mult. Scler. J..

[131] Kratz A.L., Ehde D.M., Alschuler K.N., Pickup K., Ginell K., Fritz N.E. (2024). Optimizing Detection and Prediction of Cognitive Function in Multiple Sclerosis with Ambulatory Cognitive Tests: Protocol for the Longitudinal Observational CogDetect-MS Study. JMIR Res. Protoc..

[132] Sliwinski M.J., Mogle J.A., Hyun J., Munoz E., Smyth J.M., Lipton R.B. (2018). Reliability and Validity of Ambulatory Cognitive Assessments. Assessment.

[133] Valentine T.R., Kratz A.L. (2023). Feasibility, reliability, and validity of ambulatory cognitive tests in fibromyalgia and matched controls. J. Int. Neuropsychol. Soc..

[134] Hsu W.Y., Anguera J.A., Rizzo A., Campusano R., Chiaravalloti N.D., DeLuca J., Gazzaley A., Bove R.M. (2023). A virtual reality program to assess cognitive function in multiple sclerosis: A pilot study. Front. Hum. Neurosci..

[135] Rizzo A.A., Bowerly T., Buckwalter J.G., Klimchuk D., Mitura R., Parsons T.D. (2006). A virtual reality scenario for all seasons: The virtual classroom. CNS Spectr..

[136] Floden D.P., Hogue O., Postle A.F., Busch R.M. (2024). Validation of Self-Administered Visual and Verbal Episodic Memory Tasks in Healthy Controls and a Clinical Sample. Assessment.

[137] Patrick K.S., Chakrabati S., Rhoads T., Busch R.M., Floden D.P., Galioto R. (2024). Utility of the Brief Assessment of Cognitive Health (BACH) computerized screening tool in identifying MS-related cognitive impairment. Mult. Scler. Relat. Disord..

[138] Merlo D., Darby D., Kalincik T., Butzkueven H., van der Walt A. (2019). The feasibility, reliability and concurrent validity of the MSReactor computerized cognitive screening tool in multiple sclerosis. Ther. Adv. Neurol. Disord..

[139] Merlo D., Kalincik T., Zhu C., Gresle M., Lechner-Scott J., Kilpatrick T., Barnett M., Taylor B., Buzzard K., Darby D. (2022). Subjective versus objective performance in people with multiple sclerosis using the MSReactor computerised cognitive tests. Mult. Scler. Relat. Disord..

[140] Yam C., Merlo D., Stankovich J., Darby D., Gresle M., Kalincik T., Kilpatrick T.J., Lechner-Scott J., Taylor B., Barnett M. (2020). The MSReactor computerized cognitive battery correlates with the processing speed test in relapsing-remitting multiple sclerosis. Mult. Scler. Relat. Disord..

[141] Khaligh-Razavi S.-M., Sadeghi M., Khanbagi M., Kalafatis C., Nabavi S.M. (2020). A self-administered, artificial intelligence (AI) platform for cognitive assessment in multiple sclerosis (MS). BMC Neurol..

[142] Naghavi S., Ashtari F., Adibi I., Shaygannejad V., Ramezani N., Pourmohammadi A., Davanian F., Karimi Z., Khaligh-Razavi S.M., Sanayei M. (2023). Effect of deep gray matter atrophy on information processing speed in early relapsing-remitting multiple sclerosis. Mult. Scler. Relat. Disord..

[143] Ruano L., Branco M., Severo M., Sousa A., Castelo J., Araújo I., Pais J., Cerqueira J., Amato M.P., Lunet N. (2020). Tracking cognitive impairment in multiple sclerosis using the Brain on Track test: A validation study. Neurol. Sci..

[144] Ruano L., Severo M., Sousa A., Ruano C., Branco M., Barreto R., Moreira S., Araújo N., Pinto P., Pais J. (2019). Tracking cognitive performance in the general population and in patients with mild cognitive impairment with a self-applied computerized test (brain on track). J. Alzheimer’s Dis..

[145] Ruano L., Sousa A., Severo M., Alves I., Colunas M., Barreto R., Mateus C., Moreira S., Conde E., Bento V. (2016). Development of a self-administered web-based test for longitudinal cognitive assessment. Sci. Rep..

[146] van Dongen L., Westerik B., van der Hiele K., Visser L.H., Schoonheim M.M., Douw L., Twisk J.W.R., de Jong B.A., Geurts J.J.G., Hulst H.E. (2020). Introducing Multiple Screener: An unsupervised digital screening tool for cognitive deficits in MS. Mult. Scler. Relat. Disord..

[147] Waskowiak P.T., de Jong B.A., Uitdehaag B.M.J., Saddal S.R.D., Aarts J., Roovers A.A.M., van Oirschot P., de Groot V., Schaafsma F.G., van der Hiele K. (2024). Don’t be late! Timely identification of cognitive impairment in people with multiple sclerosis: A study protocol. BMC Neurol..

[148] Beier M., Alschuler K., Amtmann D., Hughes A., Madathil R., Ehde D. (2020). iCAMS: Assessing the Reliability of a Brief International Cognitive Assessment for Multiple Sclerosis (BICAMS) Tablet Application. Int. J. MS Care.

[149] Maubeuge N., Deloire M.S., Brochet B., Charré-Morin J., Saubusse A., Ruet A. (2022). Validation of a Brief Computerized Cognitive Assessment in Multiple Sclerosis (BCCAMS) and comparison with reference batteries. Mult. Scler. J..

[150] Costabile T., Signoriello E., Lauro F., Altieri M., Ziello A.R., D’Ambrosio A., Bisecco A., Maniscalco G., Bonavita S., Gallo A. (2023). Validation of an iPad version of the Brief International Cognitive Assessment for Multiple Sclerosis (BICAMS). Mult. Scler. Relat. Disord..

[151] Podda J., Tacchino A., Ponzio M., Di Antonio F., Susini A., Pedullà L., Battaglia M.A., Brichetto G. (2024). Mobile health app (DIGICOG-MS) for self-assessment of cognitive impairment in people with multiple sclerosis: Instrument validation and usability study. JMIR Form. Res..

[152] Scaramozza M., Chiesa P.A., Zajac L., Sun Z., Tang M., Juraver A., Bartholomé E., Charré-Morin J., Saubusse A., Johnson S.C. (2024). Konectom™ cognitive processing speed test enables reliable remote, unsupervised cognitive assessment in people with multiple sclerosis: Exploring the use of substitution time as a novel digital outcome measure. Mult. Scler. J..

[153] Pratap A., Grant D., Vegesna A., Tummalacherla M., Cohan S., Deshpande C., Mangravite L., Omberg L. (2020). Evaluating the Utility of Smartphone-Based Sensor Assessments in Persons with Multiple Sclerosis in the Real-World Using an App (elevateMS): Observational, Prospective Pilot Digital Health Study. JMIR Mhealth Uhealth.

[154] Lam K.H., van Oirschot P., den Teuling B., Hulst H.E., de Jong B.A., Uitdehaag B., de Groot V., Killestein J. (2022). Reliability, construct and concurrent validity of a smartphone-based cognition test in multiple sclerosis. Mult. Scler. J..

[155] van Oirschot P., Heerings M., Wendrich K., den Teuling B., Martens M.B., Jongen P.J. (2020). Symbol Digit Modalities Test Variant in a Smartphone App for Persons with Multiple Sclerosis: Validation Study. JMIR Mhealth Uhealth.

[156] Galati A., Kriara L., Lindemann M., Lehner R., Jones J.B. (2024). User Experience of a Large-Scale Smartphone-Based Observational Study in Multiple Sclerosis: Global, Open-Access, Digital-Only Study. JMIR Hum. Factors.

[157] Midaglia L., Mulero P., Montalban X., Graves J., Hauser S.L., Julian L., Baker M., Schadrack J., Gossens C., Scotland A. (2019). Adherence and Satisfaction of Smartphone- and Smartwatch-Based Remote Active Testing and Passive Monitoring in People with Multiple Sclerosis: Nonrandomized Interventional Feasibility Study. J. Med. Internet Res..

[158] Montalban X., Graves J., Midaglia L., Mulero P., Julian L., Baker M., Schadrack J., Gossens C., Ganzetti M., Scotland A. (2022). A smartphone sensor-based digital outcome assessment of multiple sclerosis. Mult. Scler. J..

[159] Oh J., Capezzuto L., Kriara L., Schjodt-Eriksen J., van Beek J., Bernasconi C., Montalban X., Butzkueven H., Kappos L., Giovannoni G. (2024). Use of smartphone-based remote assessments of multiple sclerosis in Floodlight Open, a global, prospective, open-access study. Sci. Rep..

[160] Woelfle T., Pless S., Wiencierz A., Kappos L., Naegelin Y., Lorscheider J. (2021). Practice Effects of Mobile Tests of Cognition, Dexterity, and Mobility on Patients with Multiple Sclerosis: Data Analysis of a Smartphone-Based Observational Study. J. Med. Internet Res..

[161] Dini M., Gamberini G., Tacchini M., Boschetti A., Gradassi A., Chiveri L., Rodegher M., Comi G., Leocani L. (2025). Development and validation of an electronic Symbol-Digit Modalities Test for remote monitoring of people with multiple sclerosis. Eur. J. Neurol..

[162] Middleton R.M., Pearson O.R., Ingram G., Craig E.M., Rodgers W.J., Downing-Wood H., Hill J., Tuite-Dalton K., Roberts C., Watson L. (2020). A Rapid Electronic Cognitive Assessment Measure for Multiple Sclerosis: Validation of Cognitive Reaction, an Electronic Version of the Symbol Digit Modalities Test. J. Med. Internet Res..

[163] Maillart E., Labauge P., Cohen M., Maarouf A., Vukusic S., Donzé C., Gallien P., De Seze J., Bourre B., Moreau T. (2020). MSCopilot, a new multiple sclerosis self-assessment digital solution: Results of a comparative study versus standard tests. Eur. J. Neurol..

[164] Tanoh I.-C., Maillart E., Labauge P., Cohen M., Maarouf A., Vukusic S., Donzé C., Gallien P., De Sèze J., Bourre B. (2021). MSCopilot: New smartphone-based digital biomarkers correlate with Expanded Disability Status Scale scores in people with Multiple Sclerosis. Mult. Scler. Relat. Disord..

[165] Seo D., So J.M., Kim J., Jung H., Jang I., Kim H., Kang D.-W., Lim Y.-M., Choi J., Lee E.-J. (2024). Digital symbol-digit modalities test with modified flexible protocols in patients with CNS demyelinating diseases. Sci. Rep..

[166] Pham L., Harris T., Varosanec M., Morgan V., Kosa P., Bielekova B. (2021). Smartphone-based symbol-digit modalities test reliably captures brain damage in multiple sclerosis. NPJ Digit. Med..

[167] Barrios L., Amon R., Oldrati P., Hilty M., Holz C., Lutterotti A. (2022). Cognitive fatigability assessment test (cFAST): Development of a new instrument to assess cognitive fatigability and pilot study on its association to perceived fatigue in multiple sclerosis. Digit. Health.

[168] Kalb R., Beier M., Benedict R.H., Charvet L., Costello K., Feinstein A., Gingold J., Goverover Y., Halper J., Harris C. (2018). Recommendations for cognitive screening and management in multiple sclerosis care. Mult. Scler. J..

[169] Benedict R.H., Duquin J., Jurgensen S., Rudick R., Feitcher J., Munschauer F., Panzara M., Weinstock-Guttman B. (2008). Repeated assessment of neuropsychological deficits in multiple sclerosis using the Symbol Digit Modalities Test and the MS Neuropsychological Screening Questionnaire. Mult. Scler. J..

[170] Morrow S., Jurgensen S., Forrestal F., Munchauer F.E., Benedict R.H. (2011). Effects of acute relapses on neuropsychological status in multiple sclerosis patients. J. Neurol..

[171] Benedict R.H., DeLuca J., Phillips G., LaRocca N., Hudson L.D., Rudick R., Multiple Sclerosis Outcome Assessments Consortium (2017). Validity of the Symbol Digit Modalities Test as a cognition performance outcome measure for multiple sclerosis. Mult. Scler. J..

[172] Benedict R.H., Cohan S., Lynch S.G., Riester K., Wang P., Castro-Borrero W., Elkins J., Sabatella G. (2018). Improved cognitive outcomes in patients with relapsing–remitting multiple sclerosis treated with daclizumab beta: Results from the DECIDE study. Mult. Scler. J..

[173] Benedict R.H., Kappos L., Miller A., Hartung H.-P., Overell J., Pei J., Dahlke F., Bernasconi C., Koendgen H., Wang Q. (2025). Cognitive effects of ocrelizumab vs interferon β-1a in relapsing multiple sclerosis: A post hoc analysis of the OPERA I/II trials. Mult. Scler. Relat. Disord..

[174] Rao S.M. (1991). Neuropsychological Screening Battery for Multiple Sclerosis.

[175] Baldassari L.E., Nakamura K., Moss B.P., Macaron G., Li H., Weber M., Jones S.E., Rao S.M., Miller D., Conway D.S. (2020). Technology-enabled comprehensive characterization of multiple sclerosis in clinical practice. Mult. Scler. Relat. Disord..

[176] Patel V.P., Shen L., Rose J., Feinstein A. (2019). Taking the tester out of the SDMT: A proof of concept fully automated approach to assessing processing speed in people with MS. Mult. Scler. J..

[177] Feinstein A., Shen L., Rose J., Cayer C., Bockus C., Meza C., Puopolo J., Lapointe E. (2023). A French version of a voice recognition symbol digit modalities test analog. Can. J. Neurol. Sci..

[178] Wishart M., Everest M.R., Morrow S.A., Rose J., Shen L., Feinstein A. (2023). Establishing the consistency of a voice recognition symbol digit modalities test analogue. Mult. Scler. J..

[179] Ross D.E., Seabaugh J., Seabaugh J.M., Barcelona J., Seabaugh D., Wright K., Norwind L., King Z., Graham T.J., Baker J. (2022). Updated review of the evidence supporting the medical and legal use of NeuroQuant^®^ and NeuroGage^®^ in patients with traumatic brain injury. Front. Hum. Neurosci..

[180] Roque D.T., Teixeira R.A.A., Zachi E.C., Ventura D.F. (2011). The use of the Cambridge Neuropsychological Test Automated Battery (CANTAB) in neuropsychological assessment: Application in Brazilian research with control children and adults with neurological disorders. Psychol. Neurosci..

[181] Lee A., Archer J., Wong C.K., Chen S.H., Qiu A. (2013). Age-related decline in associative learning in healthy Chinese adults. PLoS ONE.

[182] Abbott R.A., Skirrow C., Jokisch M., Timmers M., Streffer J., van Nueten L., Krams M., Winkler A., Pundt N., Nathan P.J. (2019). Normative data from linear and nonlinear quantile regression in CANTAB: Cognition in mid-to-late life in an epidemiological sample. Alzheimer’s Dement. Diagn. Assess. Dis. Monit..

[183] Siew S.K., Han M.F., Mahendran R., Yu J. (2022). Regression-based norms and validation of the cambridge neuropsychological test automated battery among community-living older adults in Singapore. Arch. Clin. Neuropsychol..

[184] Casaletto K.B., Umlauf A., Marquine M., Beaumont J.L., Mungas D., Gershon R., Slotkin J., Akshoomoff N., Heaton R.K. (2016). Demographically Corrected Normative Standards for the Spanish Language Version of the NIH Toolbox Cognition Battery. J. Int. Neuropsychol. Soc..

[185] McHenry M.S., Roose A., Abuonji E., Nyalumbe M., Ayuku D., Ayodo G., Tran T.M., Kaat A.J. (2023). A psychometric evaluation of the NIH Toolbox fluid cognition tests adapted for Swahili and Dholuo languages in Kenyan children and adolescents. J. Int. Neuropsychol. Soc..

[186] Kuan Y.-C., Jhang K.-M., Wang W.-F., Yeh Y.-C., Chen C.-S., Yang C.-C., Hu C.-J. (2025). Cogstate Brief Battery performance in assessing cognitive impairment in Taiwan: A prospective, multi-center study. J. Formos. Med. Assoc..

[187] Yechoor N., Towe S.L., Robertson K.R., Westreich D., Nakasujja N., Meade C.S. (2016). Utility of a brief computerized battery to assess HIV-associated neurocognitive impairment in a resource-limited setting. J. Neurovirol..

[188] Bangirana P., Sikorskii A., Giordani B., Nakasujja N., Boivin M.J. (2015). Validation of the CogState battery for rapid neurocognitive assessment in Ugandan school age children. Child Adolesc. Psychiatry Ment. Health.

[189] Niino M., Miyazaki Y., Altincatal A., Belviso N., Kanda M., Chinen I., Edwards M., de Moor C., Williams J.R., Rao S.M. (2023). Processing speed test: Results from a Japanese normative sample of healthy participants compared with a US normative sample. Clin. Neurol. Neurosurg..

